# Bayesian Model Averaging of Parametric Coalescent Models for Phylodynamic Inference

**DOI:** 10.1093/molbev/msaf297

**Published:** 2025-11-21

**Authors:** Yuan Xu, Kylie Chen, Dong Xie, Alexei J Drummond

**Affiliations:** School of Biological Sciences, University of Auckland, Auckland, Aotearoa New Zealand; Centre for Computational Evolution, University of Auckland, Auckland, Aotearoa New Zealand; School of Biological Sciences, University of Auckland, Auckland, Aotearoa New Zealand; Centre for Computational Evolution, University of Auckland, Auckland, Aotearoa New Zealand; School of Biological Sciences, University of Auckland, Auckland, Aotearoa New Zealand; Centre for Computational Evolution, University of Auckland, Auckland, Aotearoa New Zealand; School of Biological Sciences, University of Auckland, Auckland, Aotearoa New Zealand; Centre for Computational Evolution, University of Auckland, Auckland, Aotearoa New Zealand

**Keywords:** phylodynamics, Bayesian model averaging, Gompertz growth, logistic growth

## Abstract

Bayesian phylodynamic models have become essential for reconstructing population history from genetic data, yet their accuracy depends crucially on choosing appropriate demographic models. To address uncertainty in model choice, we introduce a Bayesian model averaging (BMA) framework that integrates multiple parametric coalescent models—including constant, exponential, logistic, and Gompertz growth—along with their “expansion” variants that account for non-zero ancestral populations. Implemented in a Bayesian setting with Metropolis-coupled Markov chain Monte Carlo, this approach allows the sampler to switch among candidate growth functions, thereby capturing demographic histories without having to pre-specify a single model. Simulation studies verify that the logistic and Gompertz models may require specialized sampling strategies such as adaptive multivariate proposals to achieve robust mixing. We demonstrate the performance of these models on datasets simulated under different substitution models, and show that joint inference of genealogy and population parameters is well-calibrated when properly incorporating correlated-move operators and BMA. We then apply this method to two real-world datasets. Analysis of Egyptian Hepatitis C virus sequences indicates that models with a founder population followed by a rapid expansion are well supported, with a slight preference for Gompertz-like expansions. Our analysis of a metastatic colorectal cancer single-cell dataset suggests that exponential-like growth is plausible even for an advanced stage cancer patient. We believe this highlights that tumor subclones may retain substantial proliferative capacity into the later stages of the disease. Overall, our unified BMA framework reduces the need for restrictive model selection procedures and can also provide deeper biological insights into epidemic spread and tumor evolution. By systematically integrating multiple growth hypotheses within a standard Bayesian setting, this approach naturally avoids overfitting and offers a powerful tool for inferring population histories across diverse biological domains.

## Introduction

Understanding how biological systems change over time is central to many questions in biology, from population-level dynamics (Fisher 1930) to macroevolutionary patterns (Mayr 1942). In evolutionary biology, such insights help clarify the processes underlying speciation events (Pyron and Burbrink 2013; Stadler 2013), while in epidemiology, they illuminate the dynamics of rapidly evolving pathogens during viral outbreaks (Pybus et al. 2001; Faria et al. 2014). Population-level analyses are also increasingly important in tumor biology and somatic evolution, where cancer cell populations undergo mutation and selection (Leung et al. 2017; Lee-Six et al. 2018; Nowak et al. 2003; Williams et al. 2022). The advent of single-cell sequencing (Baslan and Hicks 2017) has transformed our ability to track these mutational events with unprecedented resolution, enabling detailed reconstructions of tumor evolutionary histories (Navin et al. 2011). A deeper understanding of population histories offers a flexible foundation for dissecting the origins, expansions, and adaptive trajectories of populations across diverse biological systems.

Phylodynamic models developed within a Bayesian framework provide a robust means to infer key dynamic parameters—such as growth rates and population sizes—from polymorphic gene sequence data. Approaches range from birth-death branching processes (Stadler et al. 2012, 2013; Gavryushkina et al. 2014; Kühnert et al. 2014) to Kingman’s coalescent theory (Kingman 1982; Griffiths and Tavaré 1994; Wakeley 2009), each enabling rigorous reconstruction of historical population dynamics. Among these, coalescent-based models have emerged as standard tools in population genetics (Griffiths 1980; Hudson 1990; Nordborg et al. 2001), simulating how changing population sizes shape genealogical relationships in randomly sampled lineages (Kingman 1982).

Classical coalescent theory initially assumed a constant population size, but subsequent extensions by Griffiths and Tavare (1994) and Donnelly and Tavare (1995) allowed for variable population sizes through integrable time-dependent functions. Rodrigo and Felsenstein (1999) further broadened these applications to data sampled at multiple time points—such as rapidly evolving pathogens or ancient DNA—thereby expanding the scope of coalescent inference.

Coalescent-based inference requires specifying a demographic model N(t), which describes changes in effective population size over time. Time is typically defined in reverse, setting the most recent sample time as t=0, with values increasing into the past. Demographic models can be non-parametric, allowing flexible adjustments of population size across multiple intervals (Strimmer and Pybus 2001; Drummond et al. 2005; Gill et al. 2016), or parametric, employing predefined mathematical functions to characterize trajectories of population growth or decline (Pybus et al. 2001; Pybus and Rambaut 2002; Volz et al. 2009).

Like other statistical modeling approaches, Bayesian methods are subject to model misspecification and uncertainty (Huelsenbeck and Rannala 2004). Only when the model assumptions accurately reflect the true dynamics of the sampled population do estimates of effective population size, recombination rate, and molecular clock rates yield meaningful interpretations. Bayesian phylogenetic analyses require a careful balance between model complexity and biological interpretability. The most appropriate model is not necessarily the most parameter-rich but rather one that captures essential features of the research hypothesis without overfitting or introducing excess bias. Given the inherent complexity of molecular evolutionary processes, all theoretical models are approximations of reality, making some degree of model misspecification nearly unavoidable (Baele et al. 2012). Thus, a reasonable goal for researchers is to select either a single model or a set of models that address specific research questions while closely aligning with the information content of the data. Bayesian model averaging (BMA) explicitly addresses model uncertainty by weighting candidate models according to their posterior probabilities. When multiple models are similarly supported by the data, averaged inferences tend to be more robust (Kass and Raftery 1995; Hoeting et al. 1999; Raftery et al. 2005). For nested model families, BMA also quantifies the relative contributions of individual parameters to explaining observed data (Posada and Buckley 2004). Overall, model averaging ensures that conclusions are driven by the cumulative evidence across the model space rather than relying on a single “best” model.

Two commonly used techniques for BMA are reversible jump Markov chain Monte Carlo (RJMCMC) and stochastic Bayesian variable selection. RJMCMC is elegant and widely used, allowing jumps across dimensions in complex model spaces (Hoeting et al. 1999; Bernardo 2008), originally introduced by Green (1995). However, RJMCMC can be challenging to implement, requiring careful specification of cross-dimensional proposals and computation of Jacobian determinants. Bayesian stochastic variable selection, although relatively less computationally efficient, is simpler to implement and has been extensively applied in Bayesian phylogenetic analyses (O’hara and Sillanpää 2009; Wu and Drummond 2011).

Previous studies have successfully applied Bayesian model averaging to clock models (Li and Drummond 2012; Zhang 2022) and site models (Bouckaert and Drummond 2017), but its application to parametric coalescent models remains untested. In this study, we extend the coalescent inference framework by incorporating a suite of parametric population growth models—constant, exponential, logistic, and Gompertz—alongside their “expansion” variants, which allow for a non-zero ancestral population size. The ancestral population size is the value that the function N(t) is bounded from below by as *t* approaches infinity into the past. These models are implemented in the BEAST2 phylogenetic software platform (Bouckaert et al. 2019). After validating the calibration and performance of these integrated models using simulated datasets, we implemented Bayesian model averaging across the composite model space using stochastic variable selection. Within our framework, if the phylogenetic tree is the primary focus, averaged phylogenetic estimates across multiple demographic models are obtained. Alternatively, if the demographic model itself is of interest, our framework provides posterior support for each candidate model given the observed data. Such an approach is particularly beneficial when demographic dynamics are uncertain, allowing us to incorporate model uncertainty directly into parameter estimation. By averaging across candidate models, including those with and without the ancestral population component, we reduce biases and increase the robustness of inferences about additional parameters, such as tree topology and branch lengths.

The remainder of this paper is organized as follows. First, we describe our implementation of growth models and BMA framework in BEAST2. We then validate our approach through simulation studies, demonstrating proper calibration and mixing. We also demonstrate the utility of our approach through analyses of two empirical datasets: a single-gene hepatitis C virus (HCV) dataset sampled from Egypt and a colorectal cancer (CRC) single-cell sequencing dataset.

## Methods

### Inference Framework

The coalescent model provides a flexible framework for relating observed genetic variation in a sample to underlying population demographic history (Hudson 1990). By describing the genealogical relationships among sampled sequences under a probabilistic model, it can accommodate any deterministic, time-varying population size function N(t) (Griffiths and Tavare 1994). Accurate estimation of N(t) is particularly important in epidemiological and population-genetic studies, where incorporating detailed prior information via a Bayesian framework can substantially improve inference accuracy.

Our phylodynamic inference is grounded in the joint posterior distribution:


(1) p(g,Q,Θ,μ∣D)∝Pr(D∣μ,g,Q)p(g∣Θ)p(Q)p(μ)p(Θ),

where *D* denotes the observed sequence alignment, *μ* is the molecular clock rate, *g* represents the phylogenetic time tree, *Q* comprises substitution model parameters, and *Θ* encodes the parameters of the population-size function N(t). The observed alignment *D* results from continuous-time Markov processes along the branches of *g* (Felsenstein 1981), and both the tree and population dynamic parameters are jointly estimated from the sequence data.

Consider a rooted binary time tree *g* estimated from *n* contemporaneously sampled sequences, with the sampling time defined as t=0. Define the coalescent times, measured backward from the present, as $0=t_{0}<t_{1}<\cdots <t_{n-1}$, and let $k_{i}=n-i+1$ represent the number of lineages extant immediately before the coalescent event at time $t_{i}$. In this framework, each coalescent event reduces the lineage count by one. For clarity, we describe everything in terms of contemporaneous sampling, but our implementation extends naturally to serial samples, and such extensions are well-established (Rodrigo and Felsenstein 1999; Drummond et al. 2002, 2003).

Suppose that $k_{i}$ lineages exist at time $t_{i}$. At this time, each of the $\left(\begin{array}{c} k_{i}\\ 2 \end{array}\right)$ distinct pairs of lineages has an instantaneous coalescent rate of $\frac{1}{N(t_{i})}$, where $N(t_{i})$ denotes the population size. Integrating this rate over time yields the cumulative coalescent intensity for a pair:


(2) $$F(t)=\int_{0}^{t}\frac{1}{N(u)}\;du.$$

The probability that no coalescent event occurs in the interval $\left(t_{i-1},t_{i}\right)$ (survival probability) is $\exp\;[-\left(\begin{array}{c} k_{i}\\ 2 \end{array}\right)(F(t_{i})-F(t_{i-1}))]$. When the effective population size is constant (i.e. N(t)≡N), the waiting time until the next coalescent event, $\Delta t_{i}=t_{i}-t_{i-1}$, is exponentially distributed with rate $\lambda_{i}=\left(\begin{array}{c} k_{i}\\ 2 \end{array}\right)/N$, recovering the original result of Kingman’s classical coalescent theory.

If we only record the waiting times $\Delta t_{1},\Delta t_{2},\ldots ,\Delta t_{n-1}$ (not which specific pair coalesced), then the probability density of the waiting times is:


(3) $$p(\Delta t|\Theta )=\prod_{i=1}^{n-1}\frac{\left(\begin{array}{c} k_{i}\\ 2 \end{array}\right)}{N(t_{i})}\;\exp\;\left[-\left(\begin{array}{c} k_{i}\\ 2 \end{array}\right)\left(F(t_{i})-F(t_{i-1})\right)\right].$$

The “waiting-time” density p(Δt∣Θ) represents the probability of observing these intercoalescent intervals under a coalescent model with population size N(t). To obtain the density of a particular ranked labeled time tree *g*, we must multiply this waiting-time density by the probability of selecting each specific pair of lineages represented by the tree at every coalescent event. Since exactly one of the $\left(\begin{array}{c} k_{i}\\ 2 \end{array}\right)$ lineage pairs coalesces at each event and all pairs are equally likely, the probability assigned to any particular pair is $\frac{1}{\left(\begin{array}{c} k_{i}\\ 2 \end{array}\right)}$. Thus, the probability density for observing a particular tree *g* becomes:


(4) $$p(g|\Theta )=p(\Delta t|\Theta )\times \prod_{i=1}^{n-1}\frac{1}{\left(\begin{array}{c} k_{i}\\ 2 \end{array}\right)}.$$

Substituting the explicit form of p(Δt∣Θ) defined earlier, this yields the simplified expression for the probability density of observing a particular ranked labeled tree *g*:


(5) $$p(g|\Theta )=\prod_{i=1}^{n-1}\frac{1}{N(t_{i})}\;\exp\;\left[-\left(\begin{array}{c} k_{i}\\ 2 \end{array}\right)\left(F(t_{i})-F(t_{i-1})\right)\right].$$

We must carefully distinguish between these two concepts of probability density. Since many different labeled topologies share the same waiting-time pattern, the combinatorial factor $\frac{1}{\left(\begin{array}{c} k_{i}\\ 2 \end{array}\right)}$ corrects for the number of ways to assign lineages to coalescent events. Especially, when performing model averaging over trees within an Markov chain Monte Carlo (MCMC) framework involving different tree priors, we must ensure to use the properly normalized tree density that contains the combinatorial factor in all of the tree priors under comparison.

Given this theoretical framework, provided that $\frac{1}{N(t)}$ is integrable over time, this coalescent density can be computed for any specified demographic function N(t) (Griffiths and Tavare 1994).

In practice, simulations often rely on inverse transform sampling to generate coalescent times from a specified N(t). Defining the inverse of the cumulative intensity function $F^{-1}(x)$ as the time *t* at which F(t)=x, uniformly distributed samples in *x*-space can be mapped to coalescent times in *t*-space, thus reconstructing historical population size trajectories from genetic data. When analytical solutions for $F^{-1}(x)$ are unavailable, numerical methods provide a viable alternative.

### Deterministic Population Functions

This study applies four mathematical growth models to phylodynamic inference: the constant population model, the exponential growth model, the logistic growth model, and the Gompertzian growth model. The latter three functions can be described in two forms: a base function, representing population dynamics in the absence of an ancestral population size ($N_{A}=0$), and an expansion function, obtained by introducing a non-zero $N_{A}$. This expansion accounts for ancestral contributions and is better suited for scenarios involving founding populations or prior population bottlenecks.

The exponential model provides a clear example. Its baseline form is given by:


$$N(t)=N_{0}e^{rt}.$$

By introducing a non-zero ancestral population size $N_{A}$, we obtain the exponential expansion function:


$$N(t)=(N_{0}-N_{A})e^{rt}+N_{A}.$$

When $N_{A}=0$, this expansion form reduces to the baseline model, indicating that the key difference lies in the incorporation of the ancestral population component.

The logistic model, characterized by parameters $N_{\max}$, *b*, and $t_{50}$, offers a sigmoidal representation of population growth. Its baseline form is:


$$N(t)=\frac{N_{\max}}{1+e^{-b(t-t_{50})}},$$

where $N_{\max}$ is the carrying capacity, *b* governs the rate of growth, and $t_{50}$ specifies the time at which the population reaches half of $N_{\max}$.

Incorporating an ancestral population size $N_{A}$ adjusts the model to:


$$N(t)=N_{A}+\frac{N_{\max}-N_{A}}{1+e^{-b(t-t_{50})}}.$$

This variant preserves the logistic shape but shifts the baseline to $N_{A}$. The parameter $t_{50}$ still denotes the time at which the population is halfway between $N_{A}$ and $N_{\max}$. Introducing $N_{A}$ thus extends the logistic model to scenarios involving non-zero ancestral populations.

The Gompertz model, characterized by an exponentially decreasing growth rate, is widely used to describe tumor growth dynamics. Among the various parameterizations proposed in the literature, the Gompertz-Laird formulation (Laird 1969) is frequently adopted. This classical representation involves three parameters: the initial population size $N_{0}$, the growth rate parameter *b*, and the carrying capacity $N_{\infty }$. Its baseline (no ancestral population) form is


$$N(t)=N_{0}\exp\;\left(\log\;\left(\frac{N_{\infty }}{N_{0}}\right)(1-\exp\;(-bt))\right).$$

To account for a non-zero ancestral population, we introduce a Gompertz expansion:


$$\begin{array}{rl} N(t) & =(N_{0}-N_{A})\exp\;\left(\log\;\left(\frac{N_{\infty }-N_{A}}{N_{0}-N_{A}}\right)\left[1-\exp\;(-b\;t)\right]\right)\\ & \;+N_{A}, \end{array}$$

which reduces to the baseline form if $N_{A}=0$. Here, $N_{A}$ shifts the growth trajectory upward so that the population grows from an existing size rather than near zero.

Although the parameters $\left\{N_{0},N_{\infty },b\right\}$ are biologically intuitive, $N_{0}$ and $N_{\infty }$ can be strongly correlated in MCMC sampling, potentially slowing convergence. To address this issue, adaptive methods such as the adaptable variance multivariate normal (AVMN) operator (Baele et al. 2017) can be employed to learn and adjust parameter correlations during the MCMC run.

Additionally, alternative parameterizations can be considered to further reduce correlation and enhance computational performance. One approach is to define $f_{0}=\frac{N_{0}}{N_{\infty }}\in [0,1]$. This ratio $f_{0}$ is dimensionless and constrained to the interval [0,1], enabling the straightforward use of a Beta prior without directly depending on the absolute sizes $N_{0}$ or $N_{\infty }$. Another approach, mirroring the logistic model, is to replace $N_{0}$ with a half-capacity time $t_{50}$ and $N_{\infty }$ such that $N(t_{50})=\frac{N_{\infty }}{2}$ in the baseline case, or $N(t_{50})=N_{A}+\frac{N_{\infty }-N_{A}}{2}$ when an ancestral size $N_{A}$ is present. This $\left\{N_{\infty },b,t_{50}\right\}$ parameterization helps align Gompertz with logistic growth, making $t_{50}$ a consistent reference time in both models. Detailed derivations of these transformations and prior considerations can be found in Supplementary S1.

**Fig. 1.**
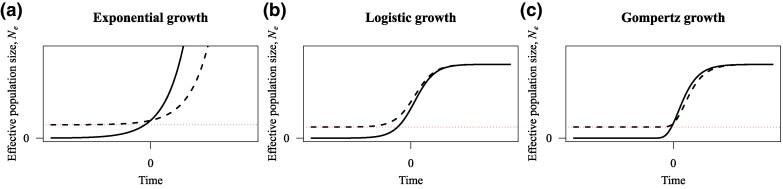
Comparison of baseline and expansion versions of demographic growth models. Dashed black curves represent the expansion model with parameter $N_{A}$, solid black curves indicate baseline models without $N_{A}$, and dotted horizontal lines represent the ancestral population size $N_{A}$. For clarity, time axes have been symmetrically extended from zero to adequately display each function’s complete behavior. (a) Exponential model, (b) Logistic model, and (c) Gompertz model.

Figure 1 illustrates the baseline and expansion forms of the exponential, logistic, and Gompertz demographic models. We define N(0) as the present-day effective population size, the horizontal axis is centered at the present (x=0): the negative half-axis (x<0) represents past time, and the positive half-axis (x>0) represents future time. For the expansion models, N(t) approaches the ancestral population baseline $N_{A}$ as one moves into the past (toward x<0); for the baseline models, we set $N_{A}=0$, so N(t) tends to 0 in the remote past.

### Model Selection and Averaging

As described in the previous method section, our Bayesian framework infers the joint posterior over the genealogy *g*, the substitution model parameters *Q*, and the demographic parameters *Θ*. We now extend *Θ* to include the discrete indicators *I* and $I_{A}$. Specifically, we define


$$\Theta =\left\{I,I_{A},N_{\max},N_{A},b,t_{50},f_{0},x,\tau \right\},$$

where *I* selects one of the growth-function families, and $I_{A}\in \{0,1\}$ determines whether a non-zero ancestral population size $N_{A}$ is included.

The prior on *Θ* includes uniform distributions for *I* and $I_{A}$, letting the data inform which demographic model is favored and whether an ancestral component is warranted.

To unify notation, we introduce $N_{\max}$ as the principal population-size parameter across all models. In the constant coalescent model, the population size is assumed to remain unchanged throughout time, whereas in the exponential model, because time is measured backward, the population function explicitly decreases from the present-day size $N_{\max}$ as time increases into the past. So the initial population size in exponential population is actually the maximum population size. And in the logistic and Gompertz models, it serves as the carrying capacity (i.e. the upper bound). Although Gompertz growth can alternatively be parameterized via $\left\{N_{0},f_{0},b\right\}$, we do not employ both parameterizations in the same analysis; thus, for consistency, we use a single symbol, $N_{\max}$, to denote the population-size parameter for all models.

Depending on the biological context, one may either unify the logistic parameter $N_{\max}$ and the Gompertz parameter $N_{\infty }$ into a single $N_{\max}$ if they are considered the same conceptual quantity, or retain them as separate parameters if their mechanistic interpretations differ. Likewise, one may choose to share a same prior for $N_{\max}$ across multiple models, or instead assign distinct priors to reflect uncertainty or variation in how each model handles the asymptotic population size. Similarly, growth-rate parameters can be treated as separate or identically distributed. When parameters in different models have the same prior, then sharing that parameter is conceptually identical to having distinct parameters, but can improve overall mixing by reducing the dimensionality of the combined model space.

To provide a unified view of how these parameters are employed across the various model variants, we summarize in Table 1 the usage of each parameter for all $\left(I,I_{A}\right)$ combinations. In the table, a tick indicates that the parameter is *used* in that model variant, whereas a empty cell indicates that the parameter is *not used*. Rows containing at least one tick indicate model variants that are included in the model averaging for the corresponding data set. Empty rows are models that are not considered for that analysis. Note that constant-size models (I=0) inherently have no expansion form (thus $I_{A}$ is irrelevant), whereas exponential, logistic, and Gompertz functions each appear in baseline ($I_{A}=0$) or expansion ($I_{A}=1$) form. For Gompertz, we use either an $f_{0}$-based or $t_{50}$-based parameterization, but not both simultaneously. Finally, the change-point parameters *x* and *τ* are used only when the selected demographic model is piece-wise, i.e. in those analyses of the empirical HCV data where a constant–exponential–constant (C–E–C) or exponential-to-constant trajectory is considered.

**Table 1:** Parameter usage across different model configurations

	HCV									L86						
Model	*I*	$I_{A}$	$N_{A}$	$N_{\max}$	*b*	$t_{50}$	$f_{0}$	*x*	*τ*	*I*	$I_{A}$	$N_{A}$	$N_{\max}$	*b*	$t_{50}$	$f_{0}$
Constant										0			✓			
Exponential(baseline)	0	0		✓	✓					1	0		✓	✓		
Exponential(expansion)	0	1	✓	✓	✓					1	1	✓	✓	✓		
Logistic(baseline)	1	0		✓	✓	✓				2	0		✓	✓	✓	
Logistic(expansion)	1	1	✓	✓	✓	✓				2	1	✓	✓	✓	✓	
Gompertz_f0(baseline)										3	0		✓	✓		✓
Gompertz_f0(expansion)										3	1	✓	✓	✓		✓
Gompertz_t50(baseline)	2	0		✓	✓	✓										
Gompertz_t50(expansion)	2	1	✓	✓	✓	✓										
Exp-to-Cons(baseline)	3	0		✓	✓			✓								
Exp-to-Cons(expansion)	3	1	✓	✓	✓			✓								
Cons–Exp–Cons	4			✓	✓			✓	✓							

This table illustrates which parameters in $\left\{I,I_{A},N_{\max},N_{A},b,t_{50},f_{0},x,\tau \right\}$ are active (ticks) or inactive (empty) under each combination of the discrete indicators $\left(I,I_{A}\right)$. For illustration, we list the models used in the HCV and L86 analyses, respectively.

We will employ these models in both the HCV and L86 applications (see below), but present this consolidated table here so that the reader can see the complete set of parameter configurations at a glance before we move to real-data analyses. A complete list of model-specific parameters and their prior is provided in Supplementary Tables S2.1 to S2.3. For details of the implementation, please refer to the scripts in Supplementary Codes S.1 and S.2.

Although these indicators can be updated via standard MCMC steps, high-dimensional or multimodal posterior distributions can challenge efficient mixing and increase the risk of becoming trapped near local maxima. To mitigate these issues, we additionally employ Metropolis-coupled MCMC (MC^3^) (Geyer 1991), which runs multiple chains in parallel at different temperatures, together with an adaptive strategy for tuning temperature differences (Müller and Bouckaert 2020). This approach helps maintain effective acceptance rates and improves overall mixing performance.

Furthermore, we integrate a stochastic variable-selection scheme within the MCMC framework to perform Bayesian model selection and averaging. Specifically, we define:

-
I∈{1,…,N} be a categorical indicator selecting among the *N* candidate demographic model families $\left\{M_{1},\ldots ,M_{N}\right\}$, and
-
$I_{A}\in \{0,1\}$ be a binary indicator specifying whether the chosen demographic model includes an ancestral population size parameter $N_{A}$.

During MCMC sampling, we jointly explore the model space by updating both the categorical indicator *I*, the ancestral-population indicator $I_{A}$, and the corresponding parameter sets $\omega_{i}$ (which include $N_{A}$ when $I_{A}=1$). This integrated approach enables not only explicit model selection but also supports BMA.

The posterior over the combined model space M is:


$$p(M_{i}|D)=\frac{\;p(D|M_{i})p(M_{i})}{\sum_{\;j=1}^{N}p(D|M_{j})p(M_{j})},$$

where $p(D|M_{i})$ represents the marginal likelihood of the data *D* under model $M_{i}$, obtained by integrating over the prior of the model-specific parameters:


$$p(D|M_{i})=\int_{\Theta_{i}}p(D|\Theta_{i},M_{i})p(\Theta_{i}|M_{i})\;d\Theta_{i}.$$

We assign equal prior probabilities to all candidate models, setting $p(M_{1})=p(M_{2})=\cdots =p(M_{N})=1/N$. Additionally, we assign a uniform prior to the binary ancestral-population indicator $I_{A}$, specifying $p(I_{A}=0)=p(I_{A}=1)=1/2$. These uniform priors imply no initial preference for any model or inclusion of the ancestral size parameter before observing the data. Consequently, posterior ratios directly correspond to Bayes factors.

### Model Validation and Simulations

To assess the reliability and calibration of our Bayesian coalescent models and the model-averaging approach, we conducted a series of simulation-based evaluations. A Bayesian model is considered well-calibrated if the nominal coverage probability of its credible intervals matches the actual frequency with which these intervals contain the true generating parameters. More formally, for 100 simulations and a nominal coverage 0.95, we have a mean expectation that 95 simulations will contain the true parameter within the 95% highest posterior density (HPD) intervals. To validate calibration, we first conducted targeted simulation experiments with 100 replicate datasets each for the constant-size, exponential-growth, and logistic-growth coalescent models (40 taxa, 2,000 bp, HKY substitution, strict clock). Additionally, we specifically focused on the Gompertz model, which includes multiple parameterizations. After confirming that each of these demographic models individually passed the calibration tests, we subsequently evaluated whether our BMA framework maintains robust inference under these complex demographic scenarios.

We used the LPhyStudio and LPhyBEAST packages within the LinguaPhylo framework (Drummond et al. 2023) to simulate data under each demographic model, generating 100 replicate datasets per model. To vary the amount of phylogenetic signal, we considered two sample sizes (n=16 and n=40) and two sequence lengths (200 bp vs. 2,000 bp). This creates settings with lower or higher evolutionary information, providing a more stringent test of parameter estimation and model recovery.

We explored two substitution models, JC69 and GT16 (Jukes and Cantor 1969; Chen et al. 2022; Kozlov et al. 2022). JC69, with uniform rates and nucleotide frequencies, served as a baseline scenario, whereas GT16 for diploid genotypes with a Dirichlet prior on genotype frequencies and substitution rates, emulating greater complexity often seen in somatic evolutionary processes.

All simulated datasets were analyzed in BEAST2 using MCMC with sufficiently long run times to ensure convergence (monitored via ESS values and trace plots). When strong correlations among parameters were detected (e.g. in Gompertz models), we introduced additional sampling operators (e.g. up–down operators) to facilitate mixing and faster convergence. Table 2 summarizes the key parameters and their prior distributions for the logistic and Gompertz models.

**Table 2:** Prior distributions for parameters under each demographic model

	Logistic	Gompertz (t50)	Gompertz (f0)
Parameter 1	$t_{50}$	$t_{50}$	$N_{0}$
*Distribution*	LogNormal(μ=0.3, σ=0.1)	LogNormal(μ=0.3, σ=0.1)	LogNormal(μ=8, σ=0.5)
**Parameter 2**	*b*	*b*	*b*
*Distribution*	LogNormal(μ=2, σ=0.5)	LogNormal(μ=2, σ=0.5)	LogNormal(μ=−0.95, σ=0.2)
**Parameter 3**	nCarryingCapacity	$N_{\infty }$	$f_{0}$
*Distribution*	LogNormal(μ=5, σ=0.3)	LogNormal(μ=4.5, σ=0.5)	Beta(α=20, β=7)

For all LogNormal distributions, *μ* (meanlog) and *σ* (sdlog) refer to mean and standard deviation on the log-scale. For the Beta distribution, *α* and *β* are shape parameters.

**Table 3:** Mean ESS across 100 replicate simulations for the Logistic and Gompertz coalescent models

Model / Parameterization	Operator scheme	ESS			
		$N_{\max}$	*b*	$t_{50}$	$f_{0}$
Logistic ($t_{50}$)	Standard	33,173	6,083	8,374	–
Gompertz ($f_{0}$)	Standard	867	83	–	84
	Improved (up–down + AVMN)	3,393	1,524	–	1,492
Gompertz ($t_{50}$)	Standard	1,605	70	57	–
	Improved (up–down + AVMN)	3,220	176	524	–

For Gompertz we report results with the original independent proposals “Standard” and after introducing the correlated-move scheme “Improved (up–down + AVMN)”. Standard and Improved runs were executed with identical MCMC chain lengths on the same set of 100 replicate XML files; the only difference is the inclusion of the up–down and AVMN operators in the Improved configuration.

### Data Analysis

In addition to the simulation study, we applied our BMA framework to two real-world datasets: (1) Egyptian HCV sequences, and (2) single-cell genomic data from the L86 metastatic CRC study. The HCV dataset consists of an alignment of 63 HCV sequences of length 411 bp, sampled in Egypt in 1993 (Ray et al. 2000); its epidemic history has previously been explored using Bayesian skyline plot methods (Drummond et al. 2005) as well as parametric Bayesian coalescent models (Pybus et al. 2003). The L86 dataset originates from a single-cell DNA sequencing study investigating metastatic CRC, comprising single-nucleus sequencing data from matched primary tumors and liver metastases (Leung et al. 2017). The original FASTQ files are publicly available in the NCBI Sequence Read Archive.

It should be noted that recombination was not explicitly modeled in our analyses of the two empirical datasets. For the HCV dataset, we followed previous studies (Pybus et al. 2003), which noted that although recombination could potentially affect coalescent analyses (Schierup and Hein 2000; Worobey 2001), recombination events in HCV are rare, having been documented in very few cases (Kalinina et al. 2002). Thus, recombination was not considered in HCV analyses. For the CRC dataset, comprising somatic mutations from tumor cells, recombination—defined as genetic exchange between distinct cell lineages—is biologically implausible, as no known mechanism supports such exchange in human tumors. Therefore, recombination was not considered a relevant factor for the CRC phylodynamic analyses presented here.

### Parametric Posterior-Based Demographic Reconstruction

After completing each MCMC run (from real data), we reconstruct historical population sizes from the model-averaged posterior on a set of discrete time points. Each iteration of the Markov chain yields a demographic model indicator and its associated parameters, defining a specific population-size function. Once sampling is finished, we define a suitable time grid—for example, from the present to the 95% HPD upper bound of the tree height—and, for each posterior sample, compute N(t) across these lattice points. We then compile these values into a posterior distribution of population sizes for each time point. Summaries such as mean, median, and credible intervals (e.g. 95% HPD) can be derived from these distributions. Plotting these statistics against time produces a continuous population trajectory that reflects both parameter and model uncertainty: if certain parametric forms appear more often in the chain, their trajectories naturally receive greater weight in the final estimate.

### Implementation

All methods described in this study have been implemented in an open-source software package named PopFunc, which is hosted in a public GitHub repository https://github.com/LinguaPhylo/PopFunc. This repository contains the core source code, modularized components for various population growth models (including Gompertz and logistic expansions), and a plugin system compatible with our Bayesian inference framework. A step-by-step example tutorial is provided in the same repository’s README, illustrating the entire analysis workflow—from an initial demo template, to generating an MC^3^-compatible BEAST2 XML, offering a practical guide for reproducing our methods.

To ensure transparency and reproducibility, we maintain a separate repository titled PopFunc-paper https://github.com/yxu927/PopFunc-paper, where we provide all simulation scripts, configuration files, and real-data analysis pipelines in this paper. In particular, PopFunc-paper includes:

- Example scripts to replicate the simulation studies in Section 1, along with instructions for parameter settings.
- Data-processing workflows for the Egyptian HCV and L86 CRC datasets (Section 1), including raw input files and post-processing routines to generate all figures and tables.

Both repositories are released under the MIT License. The scripts and code therein are sufficient to reproduce all analyses and results reported in this paper.

## Results

### Evaluation on Simulated Datasets

We first assessed the performance of the constant-size, exponential-growth, and logistic coalescent models by monitoring MCMC convergence and parameter estimation in Tracer. Across 100 replicate simulations, the mean effective sample size (ESS) values for all parameters exceeded 200 (see Supplementary Figs. S1 to S3 for detailed calibration plots).

We then applied the same chain-length settings to the Gompertz coalescent model, testing two parameterizations. In contrast to the Logistic model, the Gompertz model exhibited lower ESS, suggesting strong parameter correlations that hinder exploration (see Supplementary Fig. S4). Further joint-marginal analyses of a representative replicate revealed strong parameter correlations, which hindered efficient exploration when using standard independent proposal operators.

To address these challenges, we introduced up–down operators and integrated the AVMN operator (Baele et al. 2017), which learns the underlying posterior correlations and adapts the proposal distribution during the MCMC run. Implementing these strategies substantially enhanced sampling efficiency for the Gompertz model, with ESS values demonstrating a marked improvement across all parameters (Table 3, Supplementary Fig. S5). In particular, after applying specialized operators, 91% to 99% of the true values fell within the estimated 95% HPD intervals, indicating a well-calibrated method. Here we show a table which summarizes the mean ESS across 100 replicate simulations, explicitly comparing the two parameterizations of the Gompertz model—parameterized by fraction parameter ($f_{0}$) or midpoint time ($t_{50}$)—before and after introducing the additional operators (AVMN + up–down operators). Results for the Logistic model are also provided separately.

Next, we examined a more complex substitution and error model, the GT16 model, which accounts for amplification and allelic dropout errors in single-cell sequencing (Chen et al. 2022; Kozlov et al. 2022). Despite the increased complexity, the combination of correlated-move operators and the AVMN operator maintained good chain mixing and ESS values (see Supplementary Fig. S6). Additionally, we performed analogous simulations for the expansion variants (non-zero ancestral population) of the exponential, logistic, and Gompertz models, which also demonstrated well-calibrated parameter estimates (see Supplementary Figs. S7 to S10).

Finally, to assess how effectively our model-averaging approach can recover the correct population function under a broader set of demographic assumptions, we conducted a comprehensive simulation study involving a total of k×100=400 datasets, where k=4 represents the number of candidate demographic families (Constant, Exponential, Logistic, and Gompertz). In each replicate, we randomly selected one of the four demographic models according to a uniform prior of 1/k, resulting in approximately 100 simulated datasets per model. Each simulated dataset comprised 40-taxon trees (500 bp per sequence), generated under a strict molecular clock and the HKY substitution model. Both baseline and expansion variants were included for each demographic family except for the Constant model, which inherently lacks an expansion form, yielding seven candidate model variants in total. All candidate models had previously passed our calibration tests (see Supplementary material for details). We specified common prior across all models for the parameters $\left\{N_{A},\;N_{\max},b,\;t_{50}\right\}$. The BMA sampler yielded well-calibrated estimates for continuous parameters, including tree height, tree length, and demographic parameters. All simulation scripts are provided in Supplementary Code S.3, and the specified priors are detailed in Supplementary Table S3.

Moreover, the BMA sampler successfully identified the true generating model with high coverage across 400 replicate analyses. Specifically, we summarize the coverage metrics for the discrete model indicator *I*. Coverage for the indicator is the proportion of replicate analyses in which the true model is included in the 95% credible set. We find that the correct model is recovered in 99.3% of replicates (only 3 out of 400 did not include the true model in the 95% credible set). Moreover, the average posterior probability assigned to the true model is approximately 0.745, and the average of the highest posterior probability across all models is around 0.81, indicating that the correct model typically receives a substantial share of posterior support. Detailed results are summarized in Table 4, and full simulation scripts and specified priors are provided in Supplementary Code S.3 and Supplementary Table S3, respectively.

**Table 4:** Calibration of the model indicator under 400 simulated datasets

Generating model	Datasets (*n*)	True in 95% credible set (*n*)	Coverage (%)
Constant	103	103	100.0
Exponential	87	87	100.0
Logistic	108	107	99.1
Gompertz	102	100	98.0
**Overall**	400	397	99.3

For each dataset, first drew the generating model from the uniform prior (P(model)=0.25), simulated an alignment, and then performed Bayesian model averaging. “True in 95 % credible set” counts the runs whose 95 % model-credible set contained the true generating model. Coverage is that count divided by the number of datasets for the given model.

### Hepatitis C Virus in Egypt

HCV is genetically diverse and constitutes a leading cause of chronic liver disease worldwide. In particular, Egypt exhibits an exceptionally high prevalence of HCV, commonly attributed to large-scale parenteral antischistosomal therapy (PAT) campaigns during the mid-twentieth century (Arthur et al. 1997; Ray et al. 2000; Habib et al. 2001). At that time, unsafe injection procedures provided an efficient route for HCV transmission, facilitating rapid viral spread among the Egyptian population (Frank et al. 2000). Pybus et al. (2003) introduced a three-phase demographic model—C–E–C—to characterize the transition from a stable HCV population size, through a phase of exponential growth concurrent with PAT, and finally returning to a high-level plateau. However, previous inferences often relied on rigid demographic assumptions imposed by fixed, piecewise population-size trajectories. Recent methodological advances now allow the simultaneous integration of phylogenetic, substitution, and demographic parameters within a Bayesian framework, enabling more flexible exploration of HCV’s historical expansion in Egypt.

We configure our BMA framework with a set of parametric and piecewise demographic functions, excluding the purely constant-size model (which was deemed biologically implausible). Specifically, we incorporate:

- Baseline and expansion variants:
  We retain logistic, Gompertz, and exponential growth functions, each optionally combined with a non-zero ancestral population ($I_{A}=1$), which introduces the parameter $N_{A}$.
- Pybus’s C–E–C model: We include the original constant–exponential–constant framework from Pybus et al. (2003).
- A new “Exponential-to-Constant” model:
  This piecewise function transitions from exponential (or exponential expansion if $I_{A}=1$) to a stable plateau at $N_{\max}$. In essence, it captures a trajectory similar to the C–E–C pattern but relies on fewer parameters. At time *τ*, the model switches from exponential growth to a constant size $N_{\max}$, thereby representing both the early rapid-rise phase and the subsequent plateau in a simplified manner.

All these models reside in a common parameter space, where $I_{A}$ toggles the presence of $N_{A}$ across exponential, logistic, and Gompertz functions, while the piecewise exponential-to-constant and C–E–C models each have their own “split time” plus $N_{\max}$. We impose a shared lognormal prior on $N_{A}$ for all expansion variants, and unify logistic/Gompertz carrying capacities under a single lognormal prior for $N_{\max}$. The growth-rate parameter *b* in Gompertz has a separate prior from that of logistic/exponential, and both logistic and Gompertz share the same prior on $t_{50}$. All these priors are made sufficiently broad, thus ensuring that the MCMC can freely explore the parameter space.

By running a Metropolis-coupled MCMC across this expanded model set, we can directly compare whether the data favor Pybus’s original C–E–C, a purely parametric function (e.g. Gompertz expansion), or the new exponential-to-constant design. For each posterior sample in the MCMC chain, we record which parametric model (*I*, $I_{A}$) was selected, extract the relevant parameters (e.g. growth rate *b*, carrying capacity $N_{\max}$, ancestral size $N_{A}$), and evaluate the population trajectory at a grid of time points. By aggregating these population-size values across all samples, we derive the posterior distributions at each time bin, from which we compute the mean, median, and 95% HPD intervals.

Table 5 presents the posterior probabilities and relative log Bayes factors (logBF) for each candidate demographic model under our BMA approach. Notably, the *Gompertz Expansion* variant attains the highest posterior probability (0.438), emerging as the best-supported model. Two additional models—*Exponential expansion to constant* and *Logistic Expansion*—had moderate support (0.328 and 0.235, respectively). In contrast, the baseline versions of these functions (e.g. *Exponential*, *Logistic*, *Gompertz*  $t_{50}$), along with the piecewise C–E–C model, had negligible support (i.e. their posteriors rounded to 0.00).

**Table 5:** Posterior probabilities and logBF for candidate models in the HCV dataset

Model	Posterior	logBF
Gompertz expansion	0.438	0.00
Exponential expansion to constant	0.328	− 0.29
Logistic expansion	0.235	− 0.62
Exponential to constant	0.000	−∞
Exponential expansion	0.000	−∞
Logistic (baseline)	0.000	−∞
Gompertz $t_{50}$ (baseline)	0.000	−∞
Exponential (baseline)	0.000	−∞
C-E–C	0.000	−∞

LogBF values are computed relative to the Gompertz expansion model, which has the highest posterior probability (0.438) and is thus designated as the reference (logBF=0).

This outcome underscores the data’s strong preference for expansion-type models—those allowing for an ancestral population or additional growth-phase flexibility—over simpler or strictly piecewise assumptions. Models assigned a posterior of 0.00 also exhibit logBF values of −∞, indicating negligible support relative to the best model. Overall, these results highlight that incorporating an early founder population and subsequent rapid growth phase is critical for capturing Egypt’s HCV epidemic history.

**Fig. 2.**
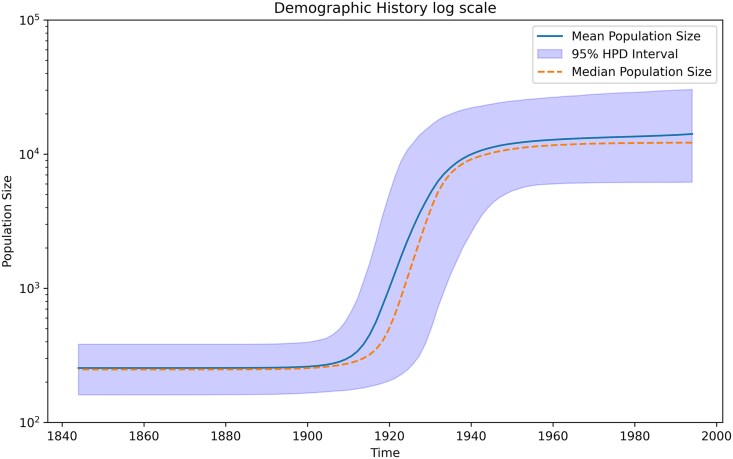
Parametric BMA Reconstruction of Egyptian HCV. The *x*-axis denotes calendar years, and the *y*-axis shows the effective population size on a log scale. The solid line is the posterior mean, the dashed line is the median, and the shaded region represents the 95% HPD interval. A pronounced rise in effective population size between the 1920s and mid-century is consistent with the era of large-scale PAT.

Figure 2 illustrates the BMA-based demographic reconstruction of HCV in Egypt, revealing a pronounced expansion during the early twentieth century and a subsequent deceleration in later decades. The solid curve denotes the mean effective population size at each time bin, the dashed curve marks the median, and the shaded region encompasses the 95% HPD interval. These results are consistent with epidemiological data indicating that widespread intravenous treatment for schistosomiasis—implemented from the 1920s to the 1950s—likely triggered the marked rise in HCV infections (Pybus et al. 2003).

### Population Dynamics in the L86 Metastatic Colorectal Cancer Dataset

To further demonstrate our model-averaging method we investigate the metastatic evolution of CRC, analyzing the L86 dataset. This dataset originates from single-cell DNA sequencing of a late-stage CRC patient who developed liver metastases (Leung et al. 2017). Despite representing an advanced clinical scenario where tumor cells have already spread from the primary (colon) to the secondary site (liver), key questions remain regarding the subclonal architecture and local population dynamics at the metastatic lesion. Focusing on the metastatic subset can reveal the growth trajectories of subclones within the metastatic niche.

Following the GT16 substitution and error model (Kozlov et al. 2022), we configured a single-run Bayesian model averaging analysis to compare seven demographic models. Specifically, we included a simpler constant model and three major growth functions (exponential, logistic, and Gompertz), each allowed to appear in either baseline ($I_{A}=0$) or expansion ($I_{A}=1$) form. Here, $N_{A}$ denotes an optional ancestral-population size—a non-zero initial number of cells present at the metastatic site at the start of the modeled epoch—and our BMA framework does not require an a priori judgment on whether an ancestral population should be included; thus, even if the use of $N_{A}$ may seem counterintuitive in cancer datasets, we keep both $I_{A}=0$ and $I_{A}=1$ models among the candidates. Note that for Gompertz, we specifically adopt the $f_{0}$-based parameterization.

Table 6 summarizes the posterior probabilities and logBF for the sampled models under this unified BMA scheme. Notably, the Exponential (baseline) model achieves the highest posterior probability (0.769), followed by Gompertz (f0) at 0.205. Together, these two account for over 97% of the total model support. In contrast, Gompertz (f0) Expansion, Logistic, and Exponential Expansion collectively account for under 3%. The Constant model had negligible posterior probability.

**Table 6:** Posterior probabilities and logBF for candidate models in the L86 metastatic CRC dataset under a single BMA analysis

Model	Posterior	logBF
Exponential	0.769	0.00
Gompertz (f0)	0.205	− 1.32
Logistic	0.0168	− 3.83
Gompertz (f0) expansion	0.00768	− 4.61
Exponential expansion	0.000791	− 6.88
Logistic expansion	0.000396	− 7.57
Constant	0.000000	−∞

LogBF values are computed relative to the best-supported model, Exponential (baseline), which thus serves as the reference (logBF=0).

**Fig. 3.**
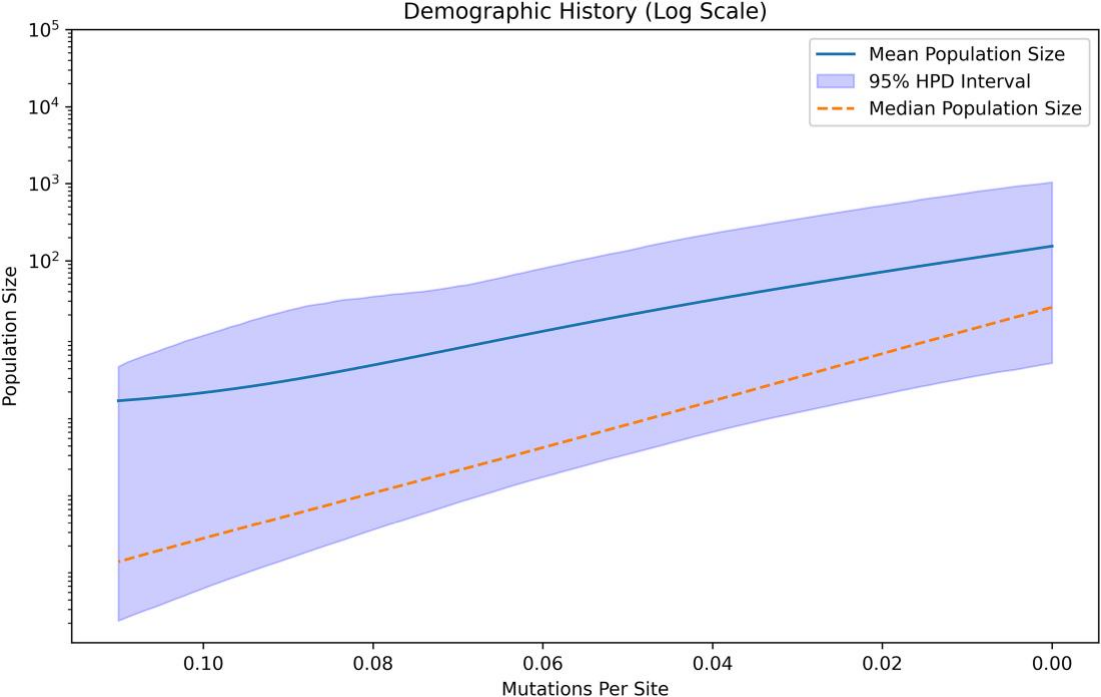
Parametric BMA Reconstruction of L86 Metastatic CRC. The *x*-axis is reversed so that time progresses from past (left) to present (right), with 0 representing the sampling year. The *x*-axis indicates relative time or approximate time since metastatic onset (in mutations per site), and the *y*-axis represents the effective population size on a log scale. The solid line is the posterior mean, the dashed line is the median, and the shaded region depicts the 95% HPD interval. Despite the advanced stage, the evidence suggests continued expansion in a near-exponential fashion.

Figure 3 presents the BMA-based reconstruction of the metastatic population trajectory, pooling samples from all candidate models (baseline and expansion). A notable feature is the apparent capacity for continued or near-exponential growth, perhaps reinforcing the idea that metastatic tumors can retain proliferative potential even at advanced clinical stages.

These results suggest that tumor cells in the metastatic lesion may still be experiencing relatively rapid growth (as reflected by the Exponential and Gompertz (f0) baseline models), contrary to the intuition that a late-stage cancer would exhibit a plateau. The data provide little support for including $N_{A}$: the Gompertz (f0) baseline performs better than its expansion form, and the Exponential baseline is likewise favored. The posterior probability of a non-zero ancestral population size is $\mathrm{Pr}(I_{A}\;|\;D)=0.0089$, indicating that an ancestral population term is unnecessary to explain these data.

## Discussion

Under the Bayesian coalescent framework, reconstructing historical population dynamics from genetic data critically depends on specifying a parametric model describing population-size trajectories over time. In practice, inference results often exhibit substantial sensitivity to the chosen demographic model; inappropriate model assumptions can lead to serious misinterpretations or even produce misleading conclusions about the true population history. In this study, we propose a BMA framework that unifies multiple demographic models into a single Bayesian joint-inference framework. By incorporating discrete model-indicator variables and employing a Metropolis-coupled MCMC approach, our method simultaneously explores multiple competing demographic hypotheses, significantly reducing the risks associated with reliance on any single model assumption. Moreover, the inclusion of flexible expansion functions coupled with binary indicator variables explicitly allows the framework to assess evidence regarding whether a non-zero ancestral population ($N_{A}$) exists, thus enhancing the flexibility and inferential capability of the modeling approach.

Simulation studies demonstrate that all models considered in this study are correctly implemented: across 100 independent simulations, the 95% HPD intervals cover the true generating parameters in approximately 95 simulations. The BMA framework shows excellent performance in model selection, successfully including the true model within the 95% credible model set in 99.3% of simulations. Furthermore, the topology and branch lengths of phylogenetic trees are accurately inferred. These results indicate that the proposed BMA framework effectively meets the needs of both identifying the true model and conducting robust inference through model averaging. Analyses of empirical datasets further validate the practicality and flexibility of this method. Analysis of the classical Egyptian HCV dataset favors the more flexible Gompertz-expansion model (posterior probability=0.44) over the historically prevalent “constant–exponential–constant” model, revealing historical population dynamics characterized by rapid initial growth from a non-zero ancestral population, followed by a gradual deceleration in the growth rate. In addition, results for the metastatic colorectal cancer dataset show the pure exponential growth model as the best-performing model (posterior probability=0.77), suggesting that the metastatic subclone maintains high proliferative activity.

Model selection is always a critical issue in statistical inference. The BMA approach proposed in this paper is an efficient and easily interpretable alternative to traditional model comparison methods. Through a single MCMC run, the relative merits of each model can be explicitly quantified using Bayes factors derived from the posterior over models. More importantly, the BMA approach allows researchers to integrate over a set of models to perform inference, thus obtaining model-averaged posterior distributions for demographic and genealogical parameters. This approach accounts for model uncertainty and mitigates the risk of model misspecification.

Finally, this study focuses on demonstrating the feasibility and flexibility of BMA in phylodynamic inference, rather than providing definitive conclusions for each empirical dataset. Therefore, our analyses of HCV and CRC datasets are primarily intended as methodological illustrations, emphasizing how the unified BMA framework effectively recovers plausible population dynamic patterns from real data, rather than attempting to establish definitive epidemiological or clinical consensus.

In future work, researchers applying our framework to specific systems may wish to evaluate the impact of different priors more systematically, exploring how robust model weights and parameter estimates remain across a range of reasonable prior settings. Such sensitivity checks can help clarify whether the resulting inferences are driven by genuine signal in the data or by implicit assumptions about population growth. Moreover, although we have demonstrated the viability of our approach with two representative datasets, it could be further extended to incorporate additional demographic structures (e.g. multi-phase or Bayesian skyline) or applied to larger single-cell genomic cohorts. In these more complex settings, the flexibility of Bayesian model averaging becomes especially advantageous, as it can reduce the risk of overfitting any single demographic function while still exploiting the strengths of each candidate model.

## Supplementary Material

msaf297_Supplementary_Data

## Data Availability

Our software, PopFunc, is publicly available at https://github.com/LinguaPhylo/PopFunc. The datasets, scripts, and analysis files supporting this manuscript are accessible at https://github.com/yxu927/PopFunc-paper. All analyses were performed using BEAST v2.7.7 (Bouckaert et al. 2019), BeastLabs v2.0.0, LPhy v1.7.0, LPhyBeast v1.3.0, and Phylonco v1.2.1 (Chen et al. 2022). Python packages used include DendroPy (Sukumaran and Holder 2010), lxml (Behnel et al. 2005), matplotlib (Hunter 2007), numpy (Harris et al. 2020), and seaborn (Waskom 2021). Additionally, the following R packages were utilized: ggtree (Yu et al. 2017), ggplot2 (Wickham and Sievert 2009), tracerR (Rambaut et al. 2018), treeSimGM (Hagen and Stadler 2018), treeio (Wang et al. 2020), expm, and ape (Paradis et al. 2019).
