# Supplementary figures and images for: Bayesian Model Averaging of Parametric Coalescent Models for Phylodynamic Inference
Source: Mol Biol Evol. 2025 Nov 21;42(12):msaf297. doi: 10.1093/molbev/msaf297 (PMC12704328; doi:10.1093/molbev/msaf297)

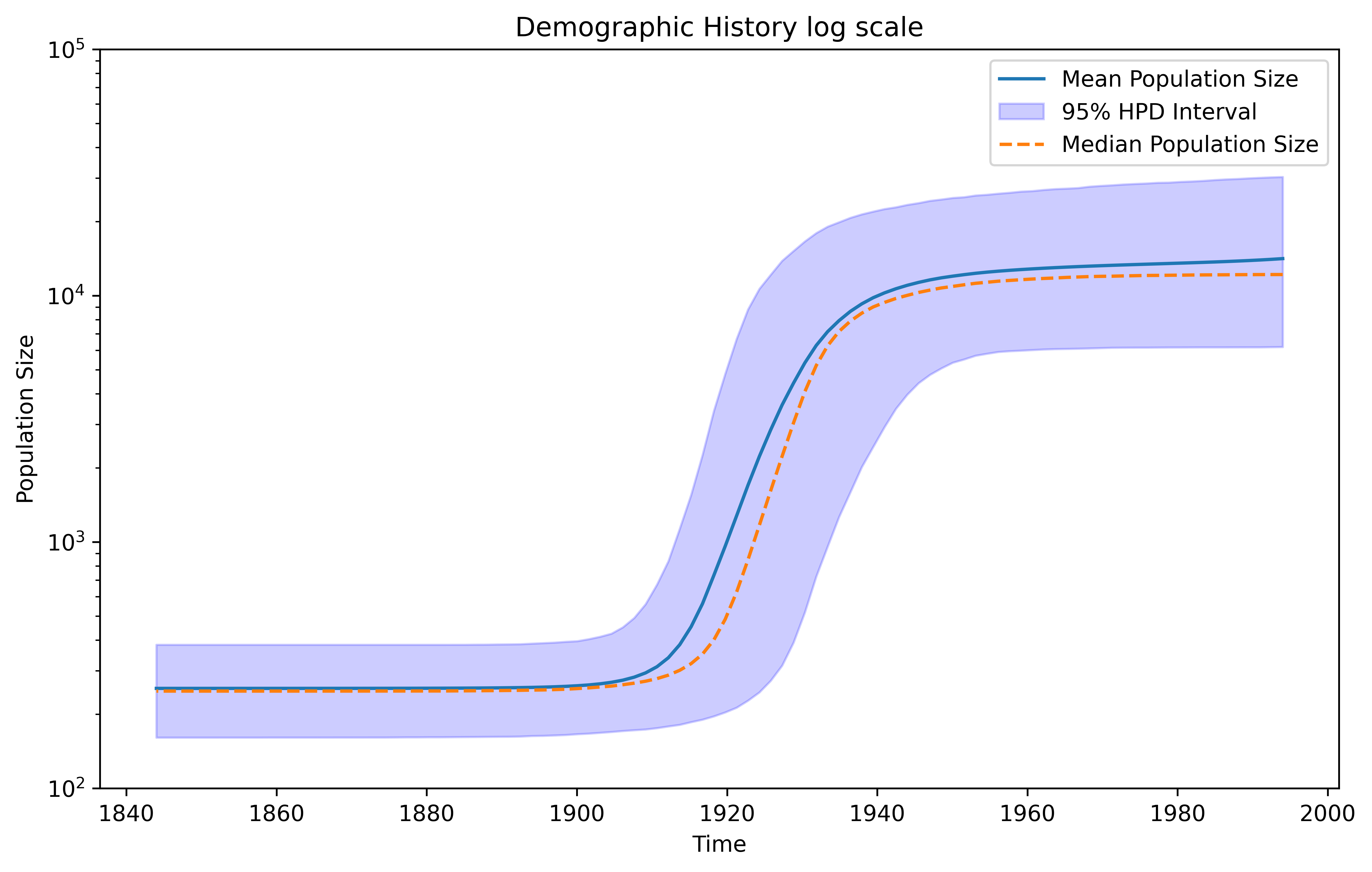

Supplement: msaf297_Supplementary_Data [file msaf297_supplementary_data.zip › figs/figs/HCV.png]

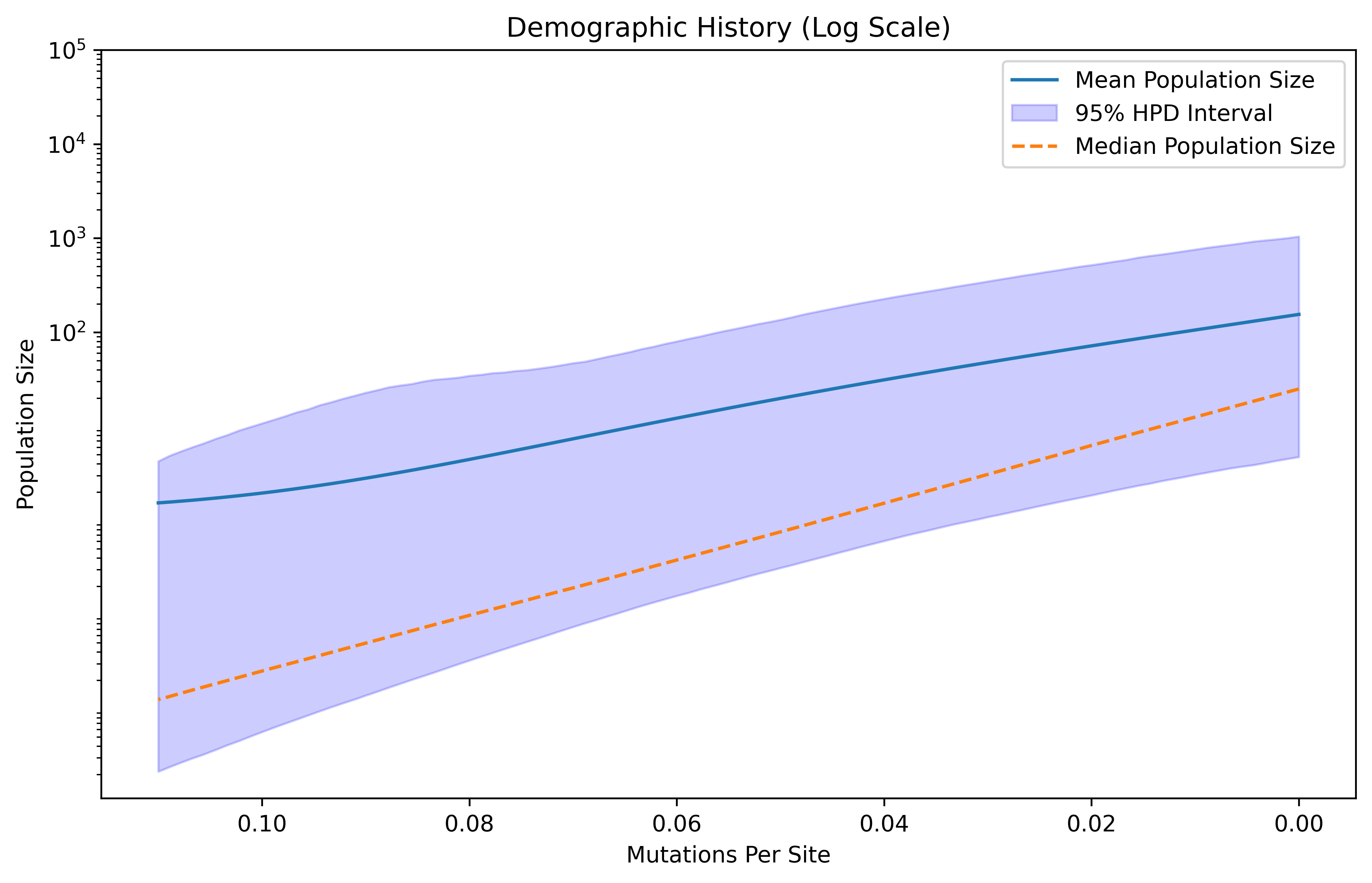

Supplement: msaf297_Supplementary_Data [file msaf297_supplementary_data.zip › figs/figs/L86.png]

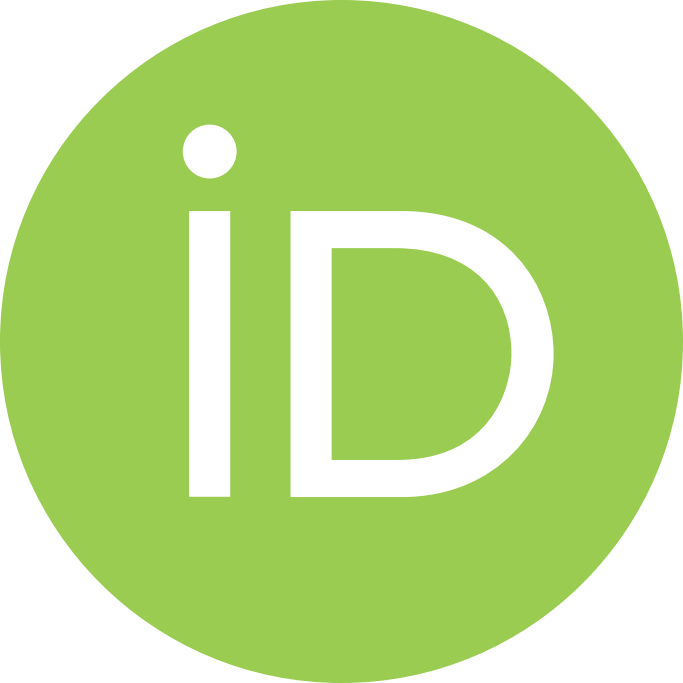

Supplement: msaf297_Supplementary_Data [file msaf297_supplementary_data.zip › figs/figs/orcid.pdf]

# Exponential growth

Effective population size,  $N_e$

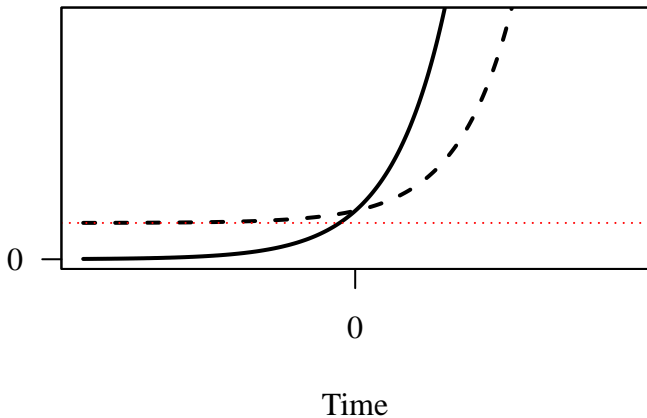

Supplement: msaf297_Supplementary_Data [file msaf297_supplementary_data.zip › figs/figs/plot/exp.pdf]

## Gompertz growth

Effective population size,  $N_e$

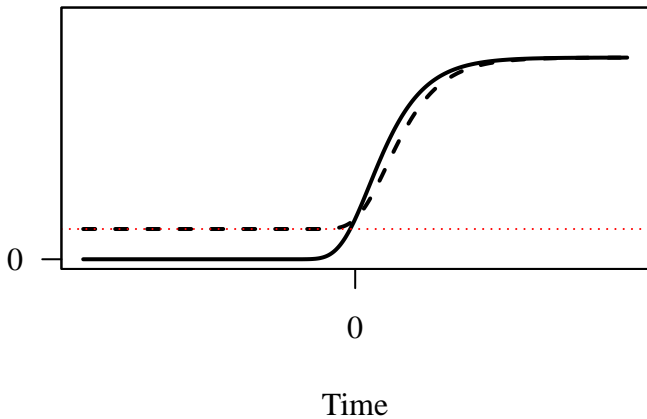

Supplement: msaf297_Supplementary_Data [file msaf297_supplementary_data.zip › figs/figs/plot/Gompertz.pdf]

# Logistic growth

Effective population size,  $N_e$

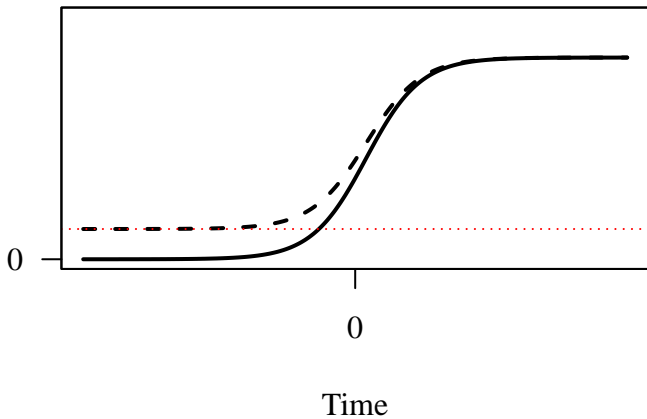

Supplement: msaf297_Supplementary_Data [file msaf297_supplementary_data.zip › figs/figs/plot/logistic.pdf]

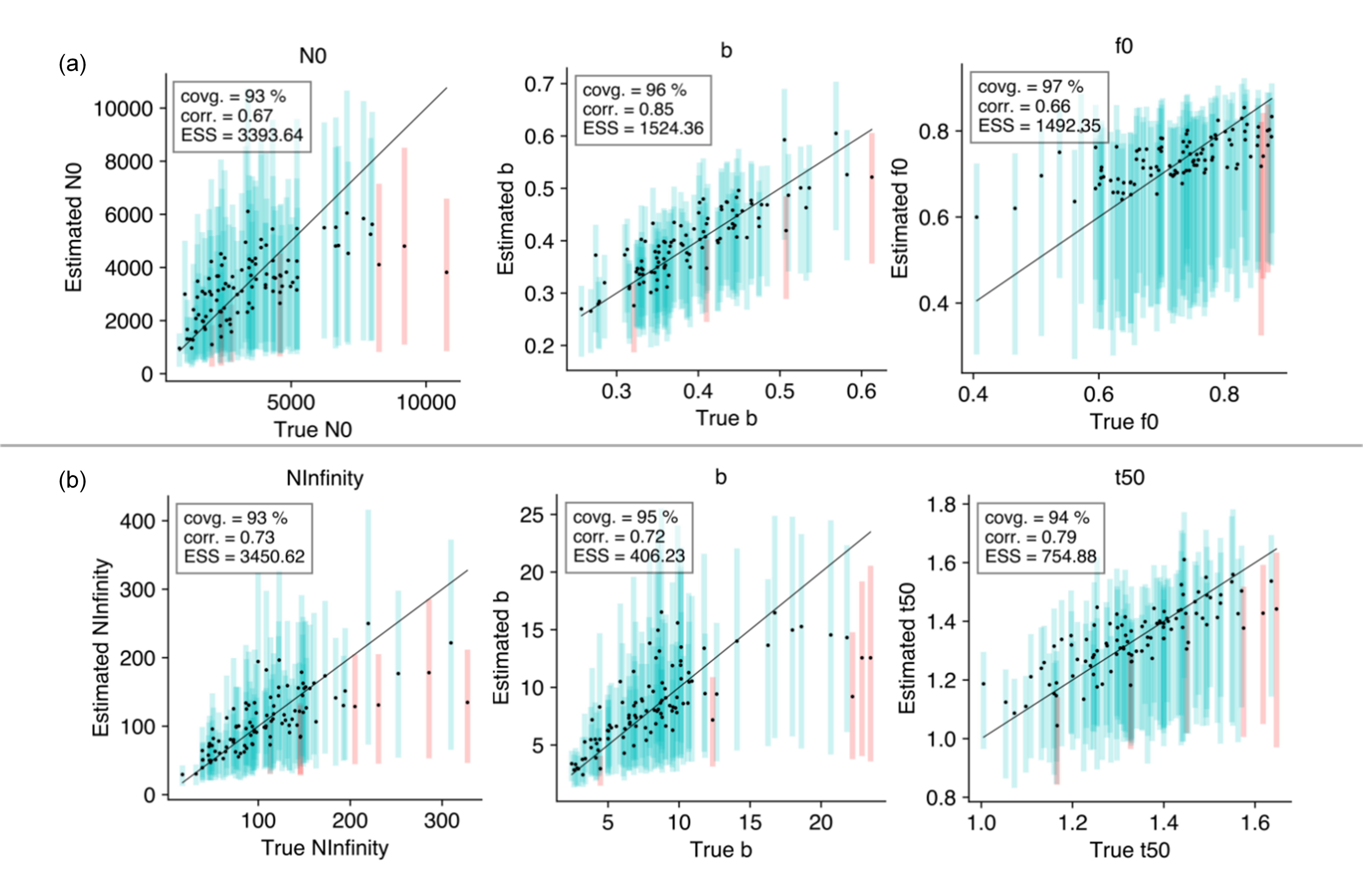

Supplement: msaf297_Supplementary_Data [file msaf297_supplementary_data.zip › Tex_LaTeX_suppl/supplement/figs/gompertz(add).png]

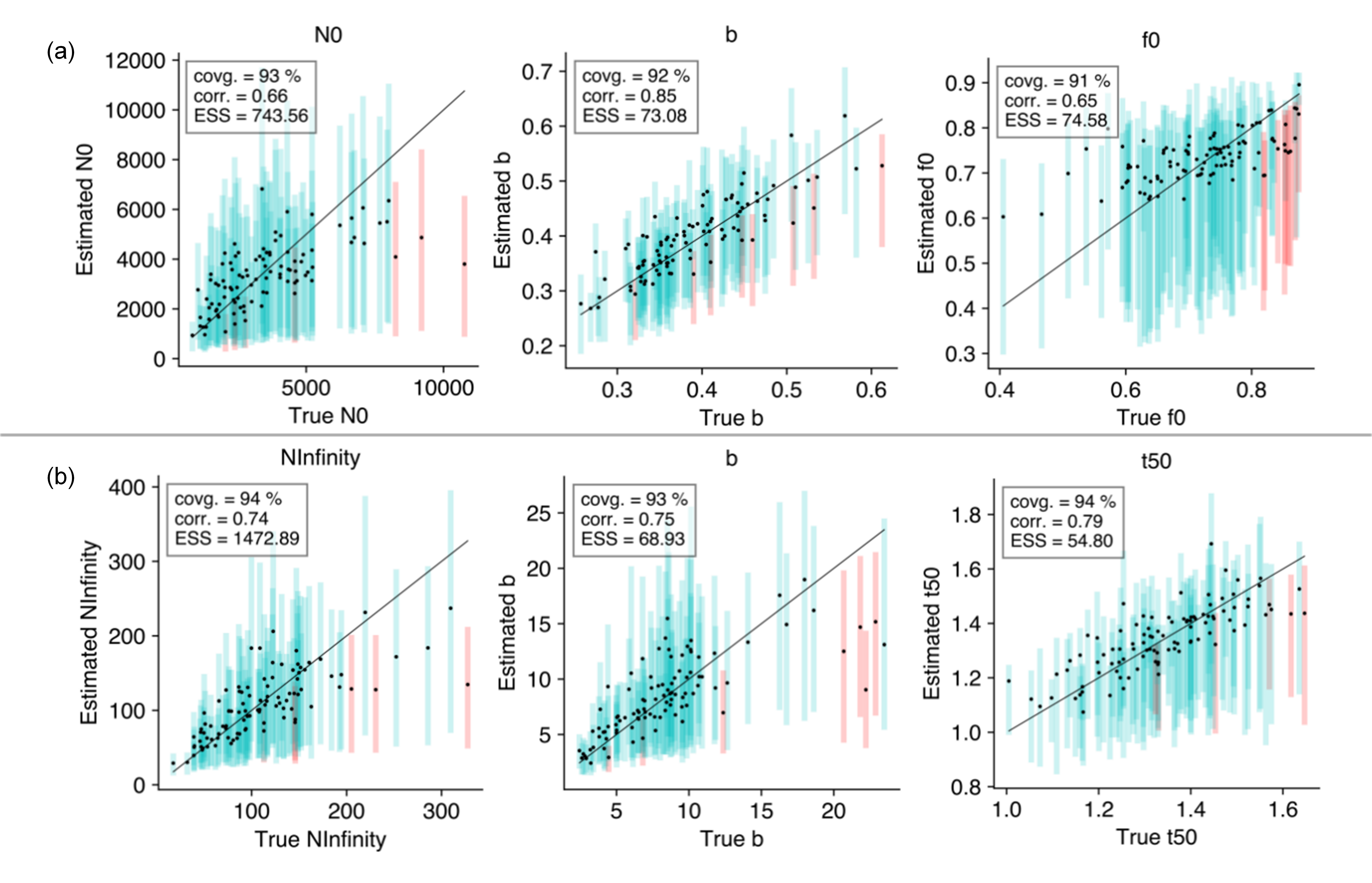

Supplement: msaf297_Supplementary_Data [file msaf297_supplementary_data.zip › Tex_LaTeX_suppl/supplement/figs/gompertz.jpg]

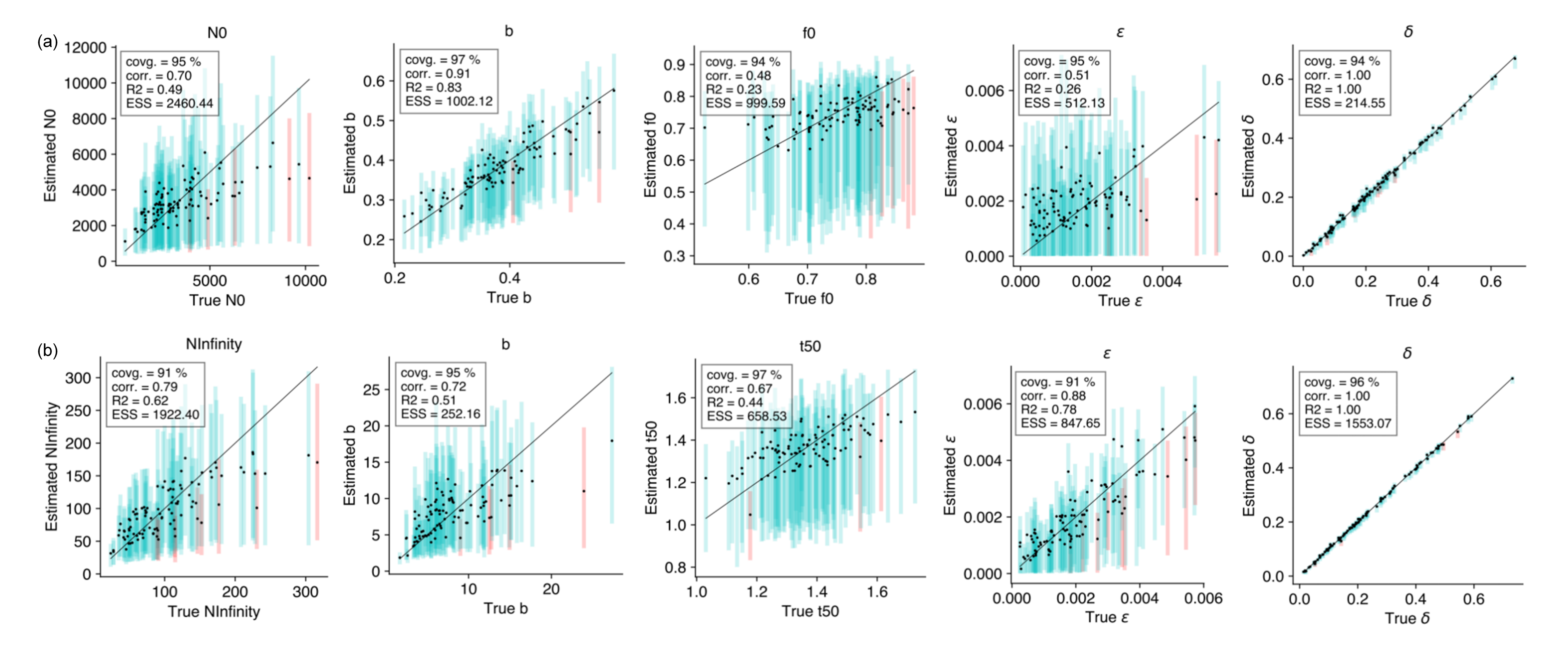

Supplement: msaf297_Supplementary_Data [file msaf297_supplementary_data.zip › Tex_LaTeX_suppl/supplement/figs/gt16.jpg]

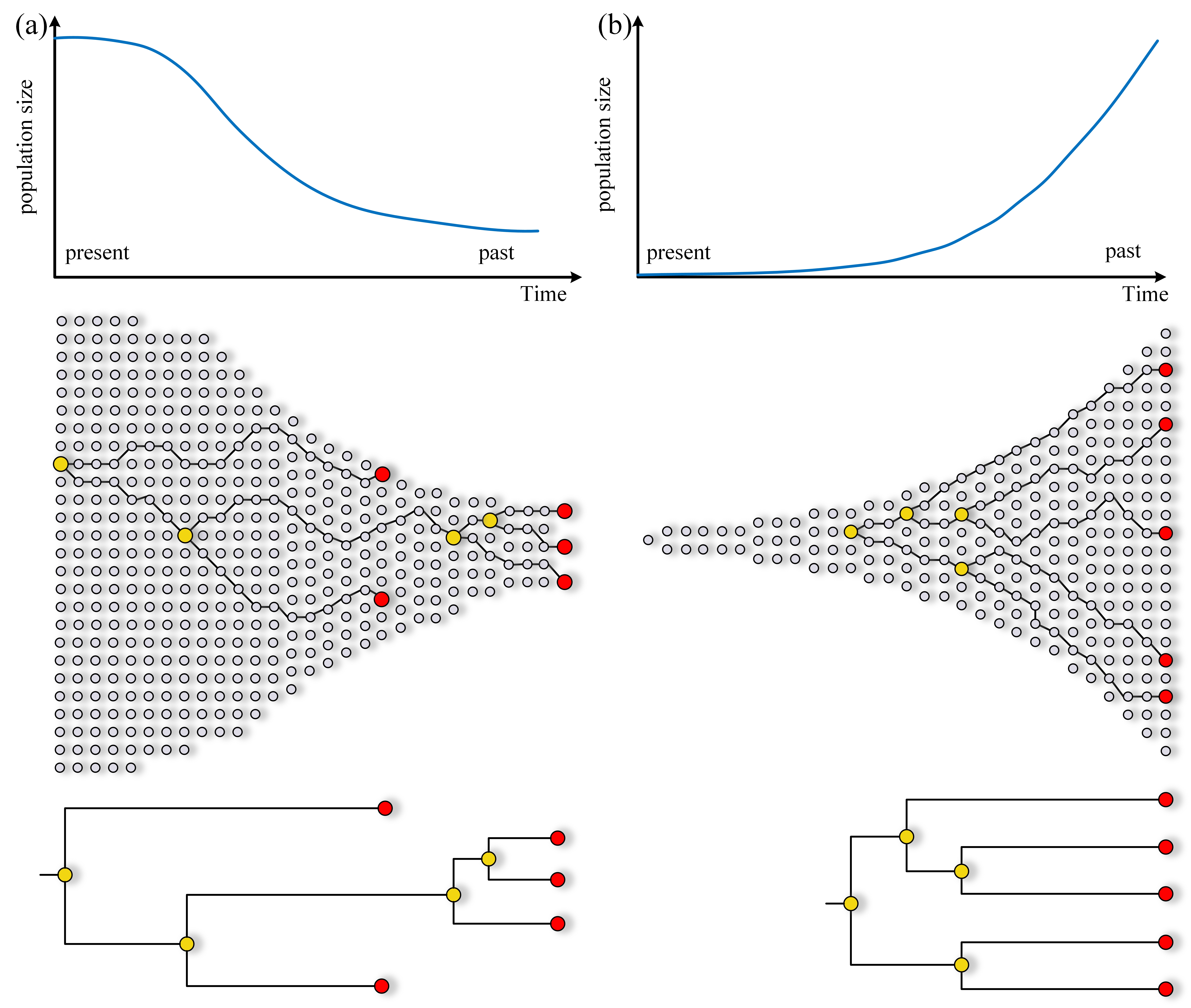

Supplement: msaf297_Supplementary_Data [file msaf297_supplementary_data.zip › Tex_LaTeX_suppl/supplement/figs/image0.png]

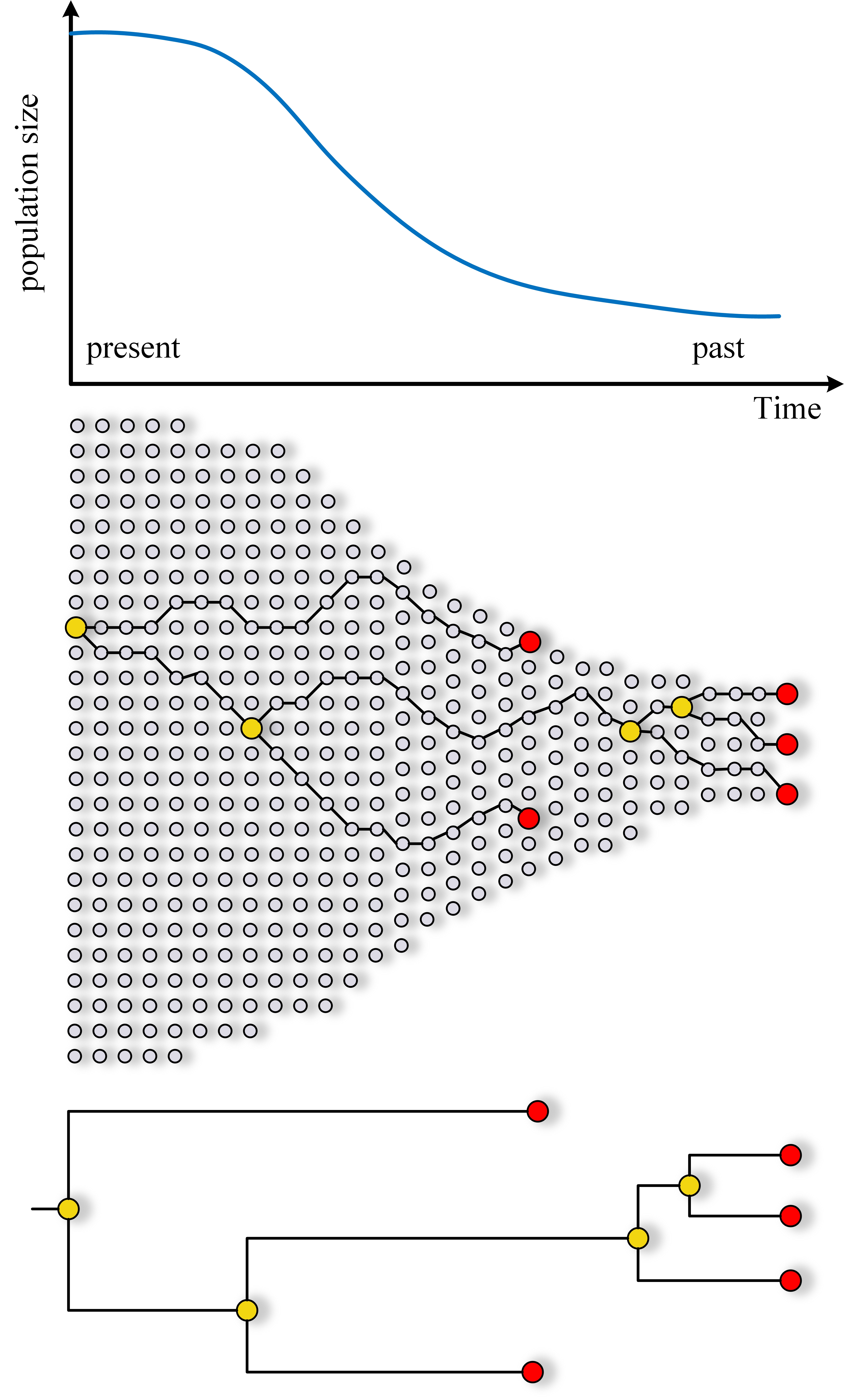

Supplement: msaf297_Supplementary_Data [file msaf297_supplementary_data.zip › Tex_LaTeX_suppl/supplement/figs/image1.png]

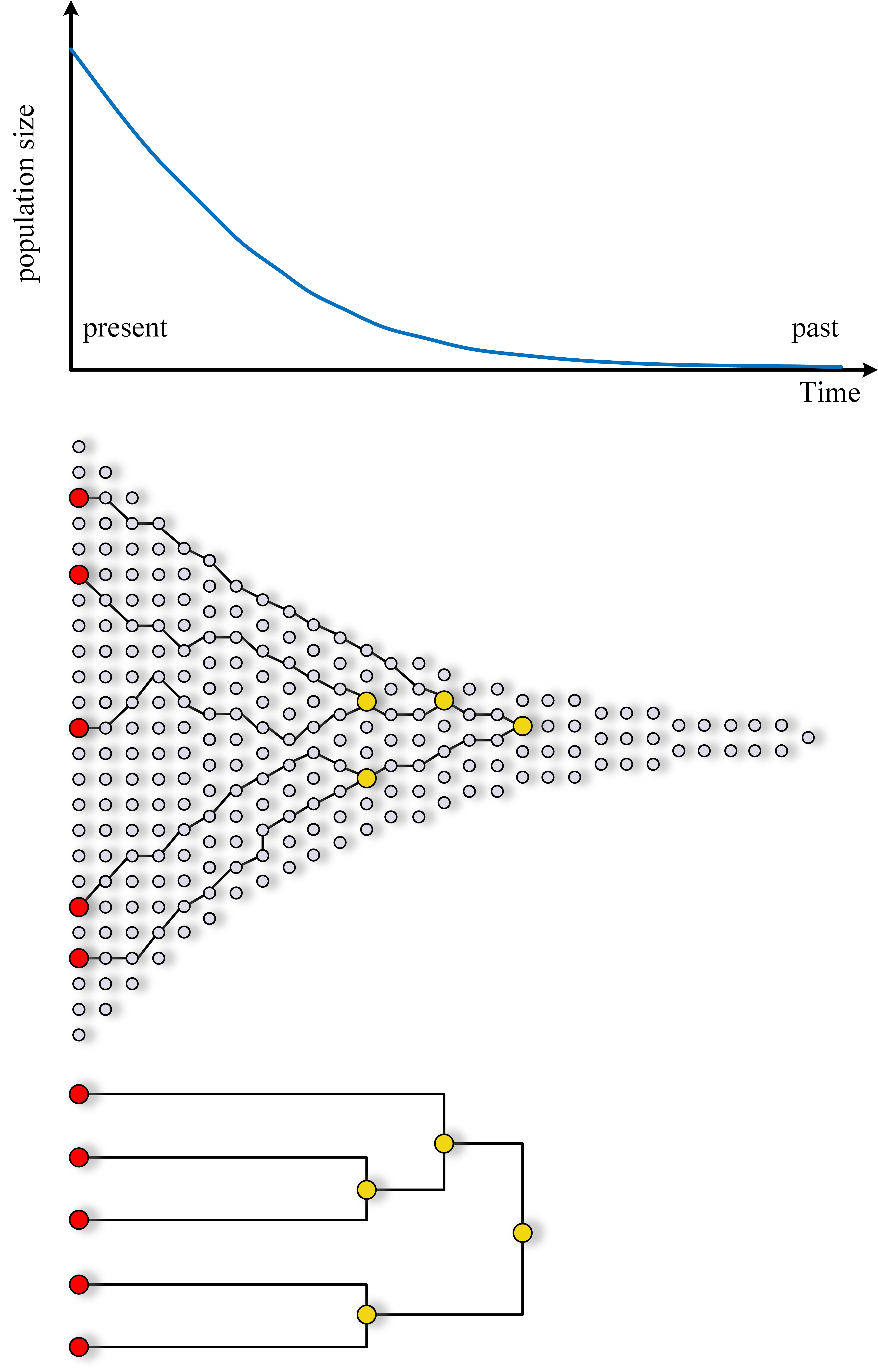

Supplement: msaf297_Supplementary_Data [file msaf297_supplementary_data.zip › Tex_LaTeX_suppl/supplement/figs/image2.png]

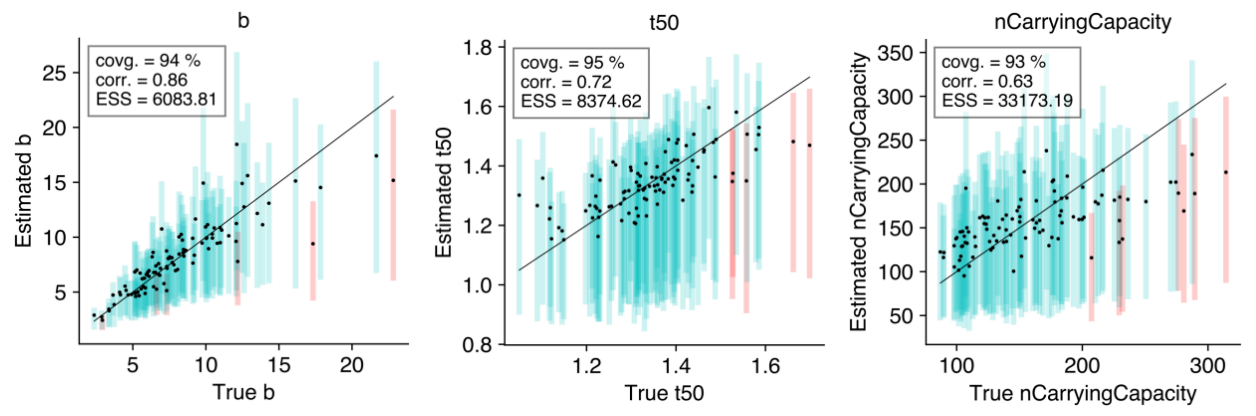

Supplement: msaf297_Supplementary_Data [file msaf297_supplementary_data.zip › Tex_LaTeX_suppl/supplement/figs/logistic.png]

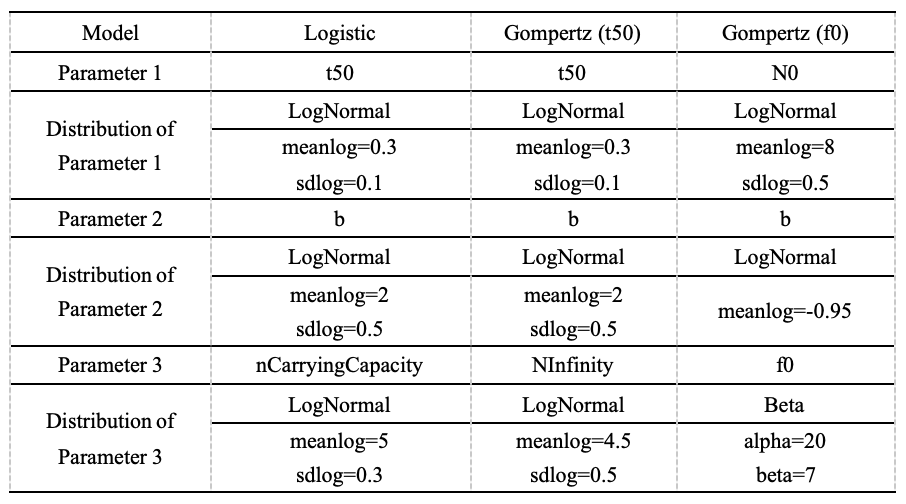

Supplement: msaf297_Supplementary_Data [file msaf297_supplementary_data.zip › Tex_LaTeX_suppl/supplement/figs/table1.png]

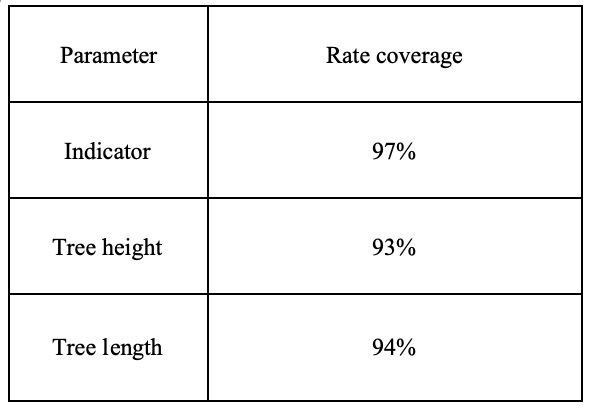

Supplement: msaf297_Supplementary_Data [file msaf297_supplementary_data.zip › Tex_LaTeX_suppl/supplement/figs/table2.png]

N0

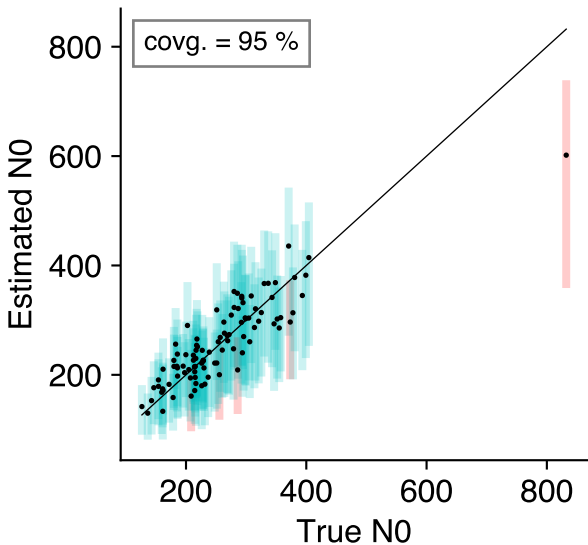

Supplement: msaf297_Supplementary_Data [file msaf297_supplementary_data.zip › Tex_LaTeX_suppl/supplement/figs/constant/n0.pdf]

# GrowthRate

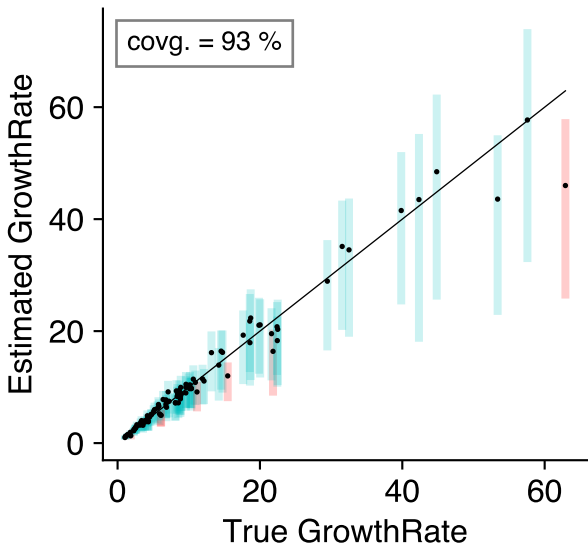

Supplement: msaf297_Supplementary_Data [file msaf297_supplementary_data.zip › Tex_LaTeX_suppl/supplement/figs/exp/growthrate.pdf]

N0

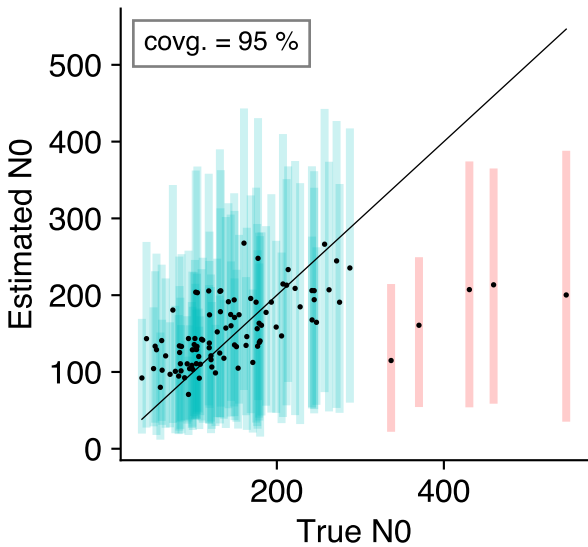

Supplement: msaf297_Supplementary_Data [file msaf297_supplementary_data.zip › Tex_LaTeX_suppl/supplement/figs/exp/n0.pdf]

# GrowthRate

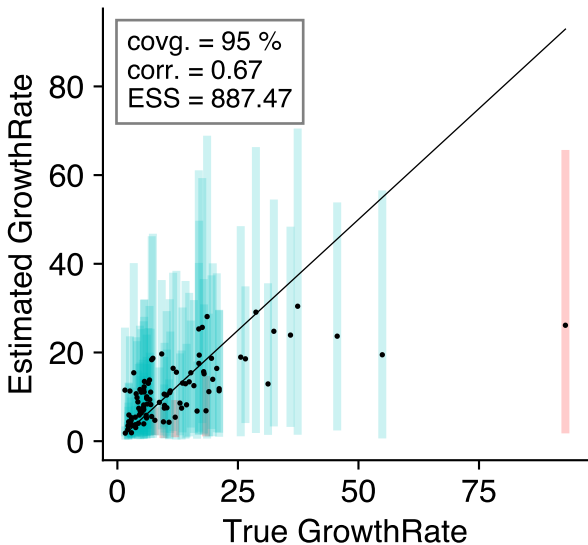

Supplement: msaf297_Supplementary_Data [file msaf297_supplementary_data.zip › Tex_LaTeX_suppl/supplement/figs/expExpansion/growthrate.pdf]

N0

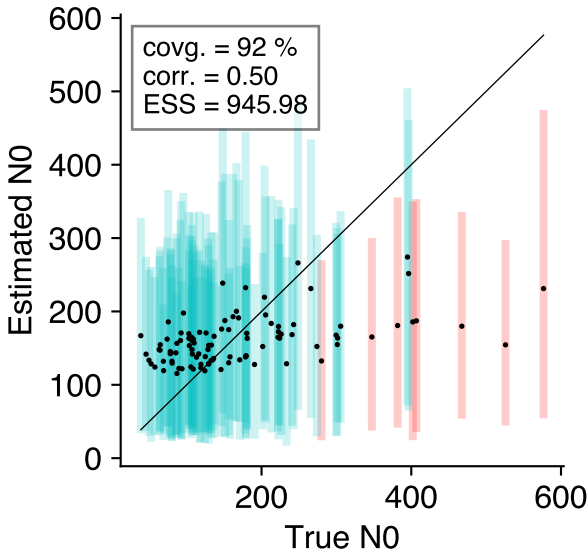

Supplement: msaf297_Supplementary_Data [file msaf297_supplementary_data.zip › Tex_LaTeX_suppl/supplement/figs/expExpansion/n0.pdf]

NA

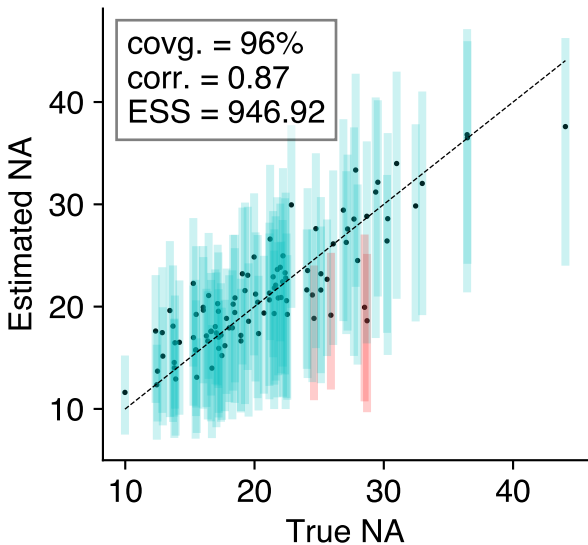

Supplement: msaf297_Supplementary_Data [file msaf297_supplementary_data.zip › Tex_LaTeX_suppl/supplement/figs/expExpansion/NA.pdf]

b

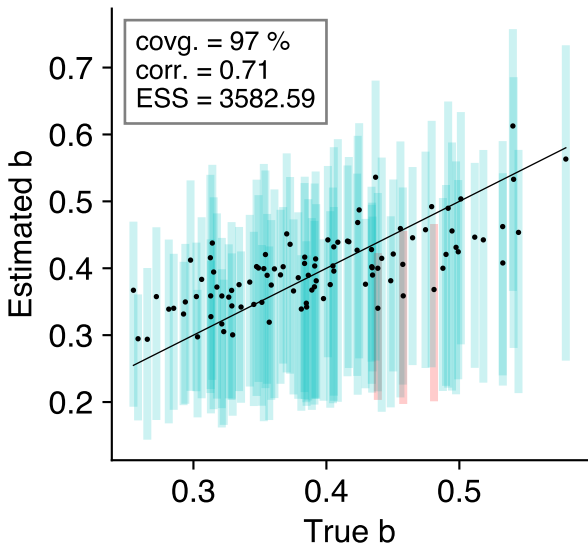

Supplement: msaf297_Supplementary_Data [file msaf297_supplementary_data.zip › Tex_LaTeX_suppl/supplement/figs/gompertzExpansion_f0/b.pdf]

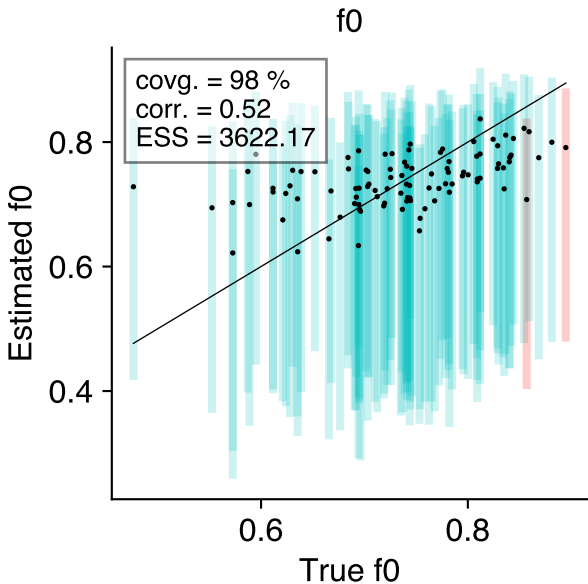

Supplement: msaf297_Supplementary_Data [file msaf297_supplementary_data.zip › Tex_LaTeX_suppl/supplement/figs/gompertzExpansion_f0/f0.pdf]

N0

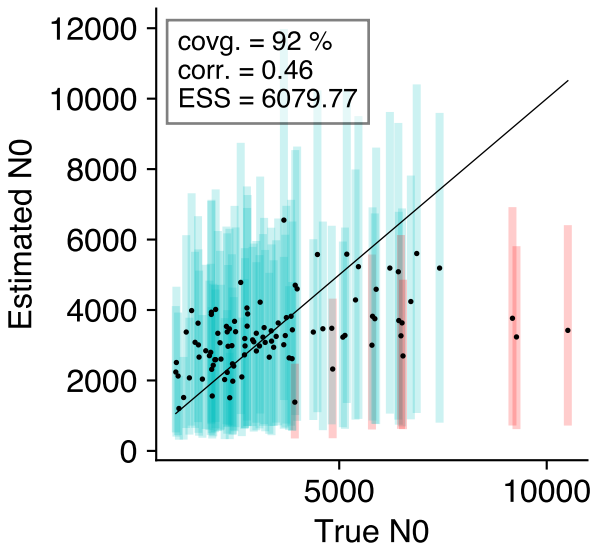

Supplement: msaf297_Supplementary_Data [file msaf297_supplementary_data.zip › Tex_LaTeX_suppl/supplement/figs/gompertzExpansion_f0/n0.pdf]

NA

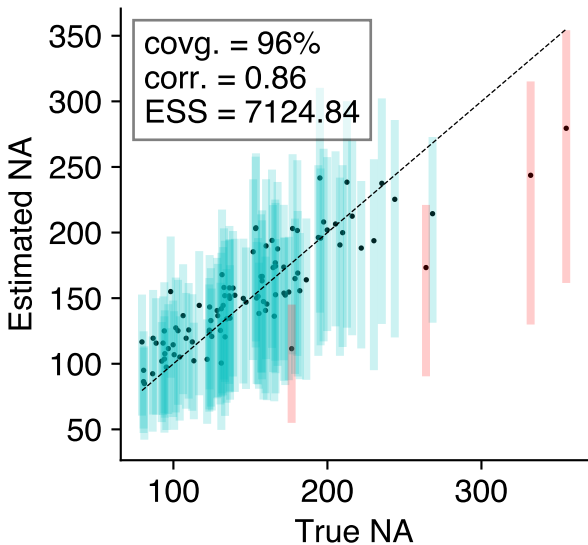

Supplement: msaf297_Supplementary_Data [file msaf297_supplementary_data.zip › Tex_LaTeX_suppl/supplement/figs/gompertzExpansion_f0/NA.pdf]

b

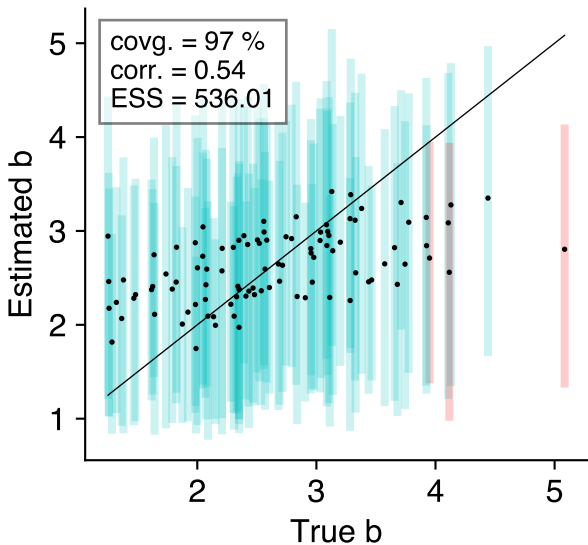

Supplement: msaf297_Supplementary_Data [file msaf297_supplementary_data.zip › Tex_LaTeX_suppl/supplement/figs/gompertzExpansion_t50/b.pdf]

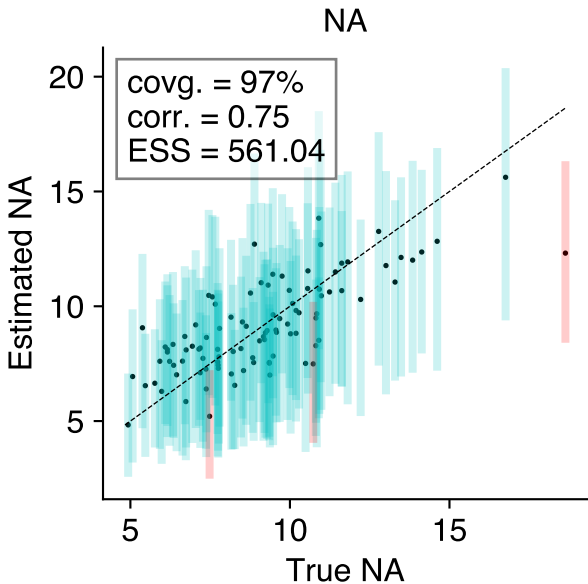

Supplement: msaf297_Supplementary_Data [file msaf297_supplementary_data.zip › Tex_LaTeX_suppl/supplement/figs/gompertzExpansion_t50/NA.pdf]

NlInfinity

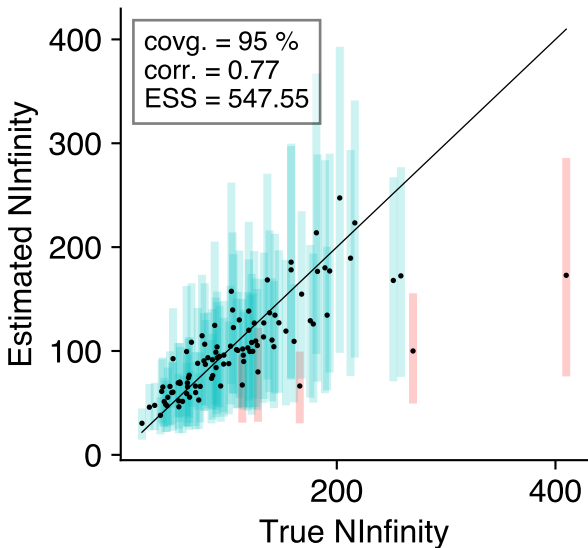

Supplement: msaf297_Supplementary_Data [file msaf297_supplementary_data.zip › Tex_LaTeX_suppl/supplement/figs/gompertzExpansion_t50/ninfinity.pdf]

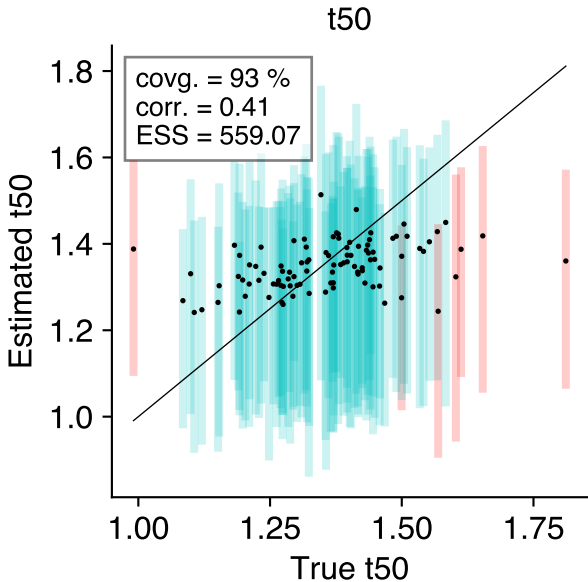

Supplement: msaf297_Supplementary_Data [file msaf297_supplementary_data.zip › Tex_LaTeX_suppl/supplement/figs/gompertzExpansion_t50/t50.pdf]

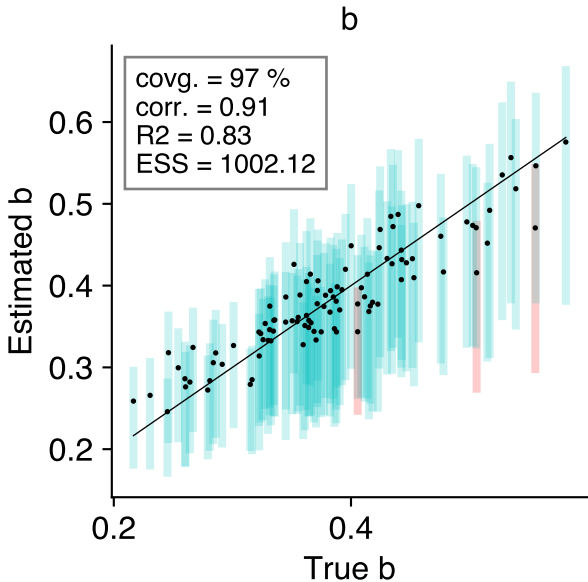

Supplement: msaf297_Supplementary_Data [file msaf297_supplementary_data.zip › Tex_LaTeX_suppl/supplement/figs/gt16/gt16-f0/b.pdf]

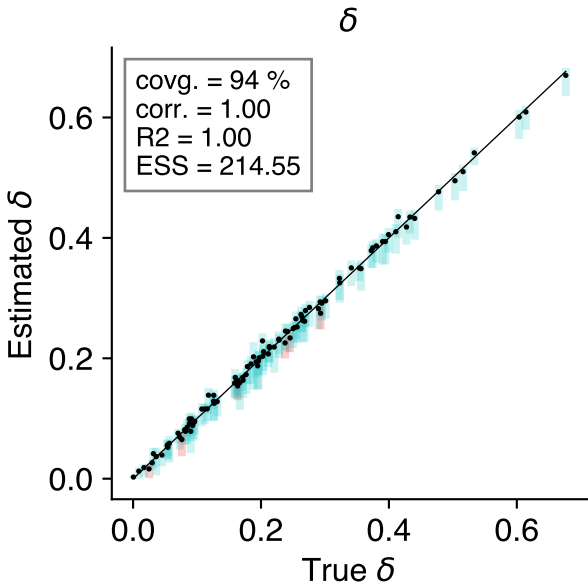

Supplement: msaf297_Supplementary_Data [file msaf297_supplementary_data.zip › Tex_LaTeX_suppl/supplement/figs/gt16/gt16-f0/delta.pdf]

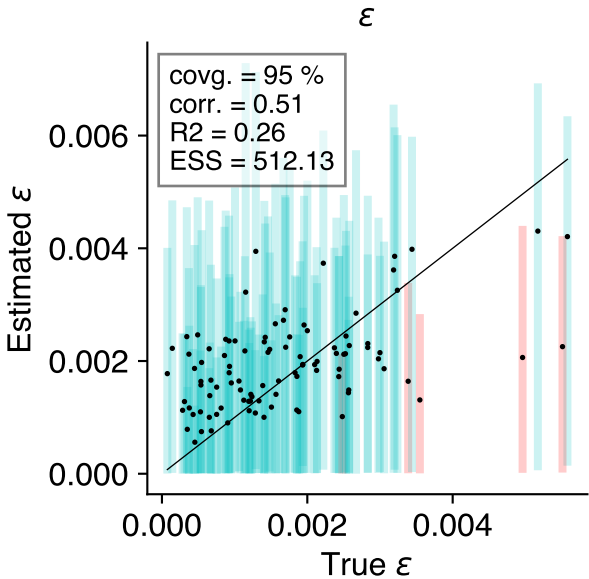

Supplement: msaf297_Supplementary_Data [file msaf297_supplementary_data.zip › Tex_LaTeX_suppl/supplement/figs/gt16/gt16-f0/epsilon.pdf]

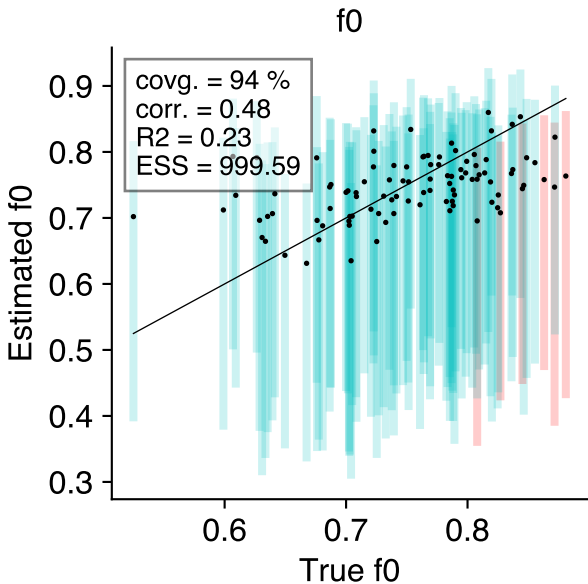

Supplement: msaf297_Supplementary_Data [file msaf297_supplementary_data.zip › Tex_LaTeX_suppl/supplement/figs/gt16/gt16-f0/f0.pdf]

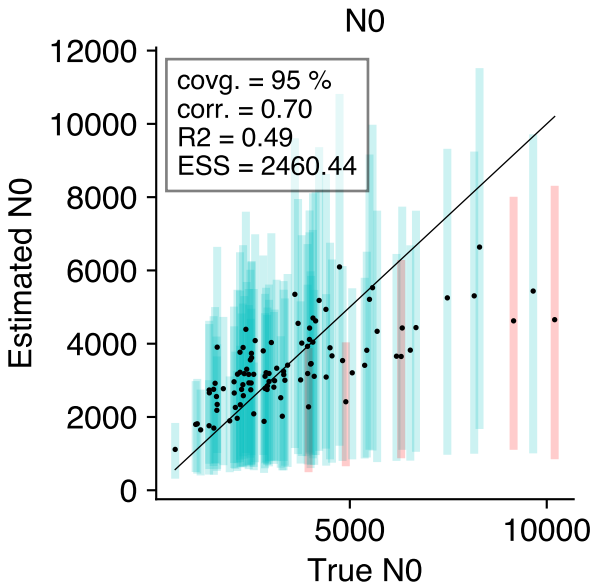

Supplement: msaf297_Supplementary_Data [file msaf297_supplementary_data.zip › Tex_LaTeX_suppl/supplement/figs/gt16/gt16-f0/n0.pdf]

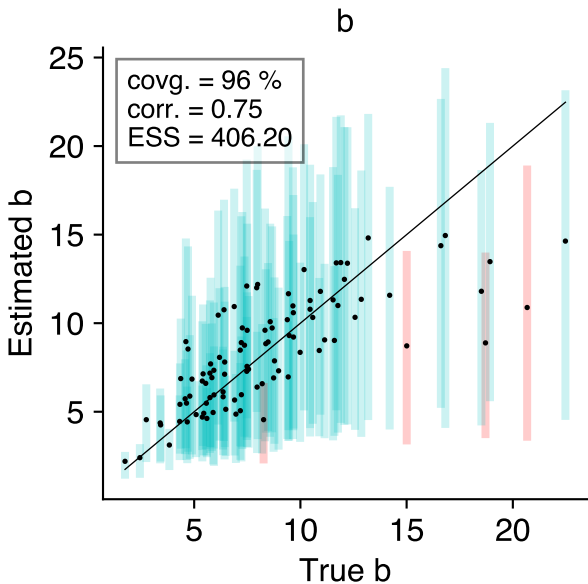

Supplement: msaf297_Supplementary_Data [file msaf297_supplementary_data.zip › Tex_LaTeX_suppl/supplement/figs/gt16/gt16-t50/b.pdf]

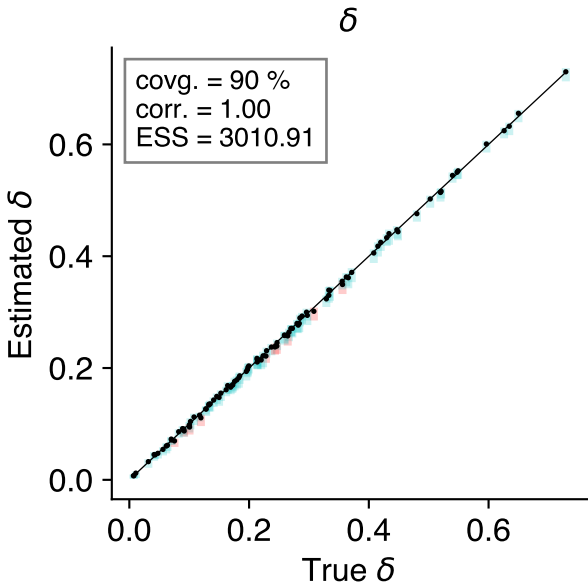

Supplement: msaf297_Supplementary_Data [file msaf297_supplementary_data.zip › Tex_LaTeX_suppl/supplement/figs/gt16/gt16-t50/delta.pdf]

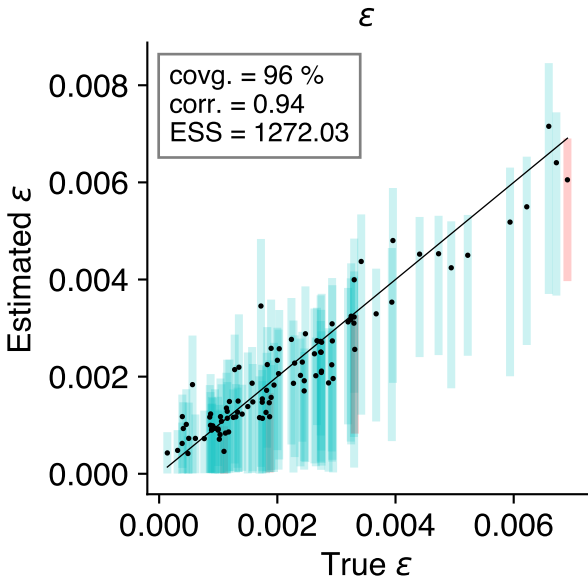

Supplement: msaf297_Supplementary_Data [file msaf297_supplementary_data.zip › Tex_LaTeX_suppl/supplement/figs/gt16/gt16-t50/epsilon.pdf]

# NInfinity

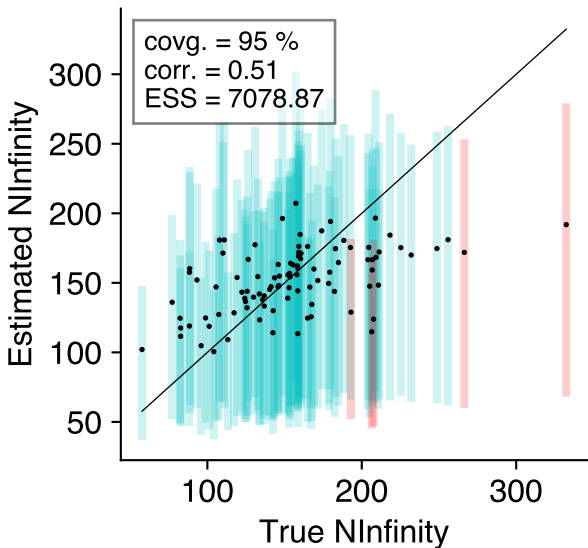

Supplement: msaf297_Supplementary_Data [file msaf297_supplementary_data.zip › Tex_LaTeX_suppl/supplement/figs/gt16/gt16-t50/Ninf.pdf]

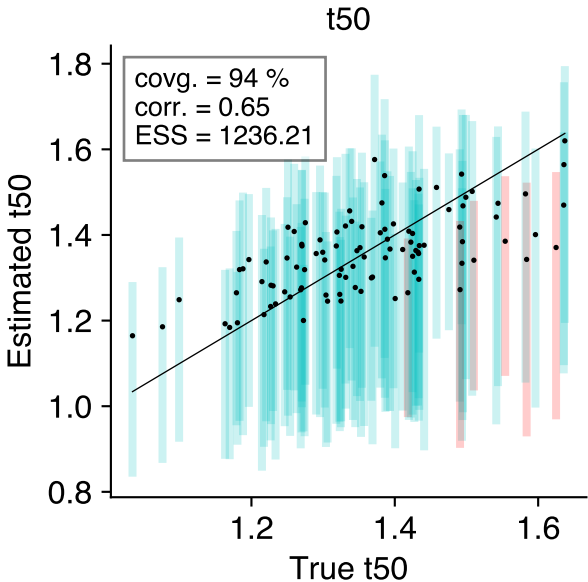

Supplement: msaf297_Supplementary_Data [file msaf297_supplementary_data.zip › Tex_LaTeX_suppl/supplement/figs/gt16/gt16-t50/t50.pdf]

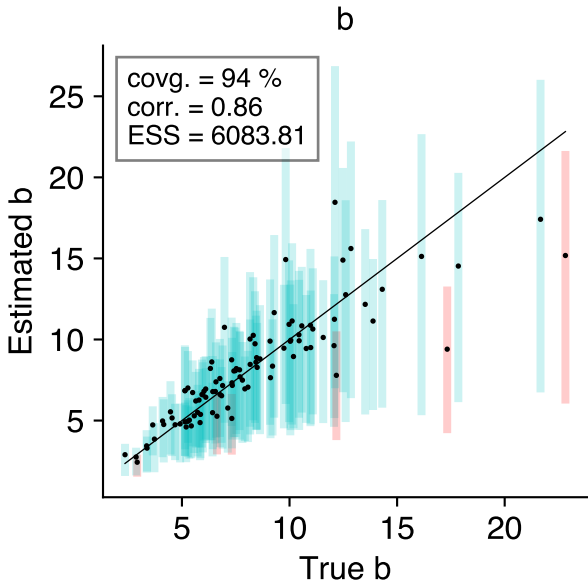

Supplement: msaf297_Supplementary_Data [file msaf297_supplementary_data.zip › Tex_LaTeX_suppl/supplement/figs/logistic/b.pdf]

# nCarryingCapacity

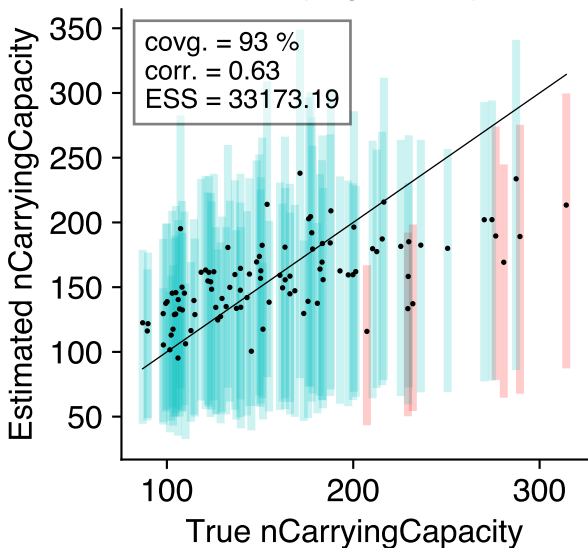

Supplement: msaf297_Supplementary_Data [file msaf297_supplementary_data.zip › Tex_LaTeX_suppl/supplement/figs/logistic/Ninf.pdf]

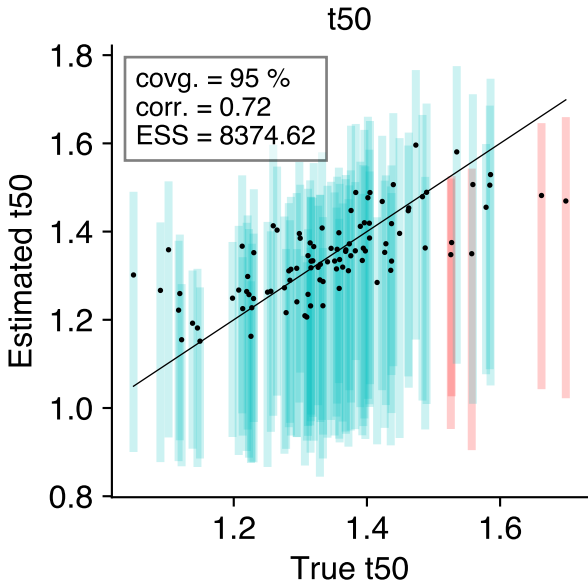

Supplement: msaf297_Supplementary_Data [file msaf297_supplementary_data.zip › Tex_LaTeX_suppl/supplement/figs/logistic/t50.pdf]

**b**

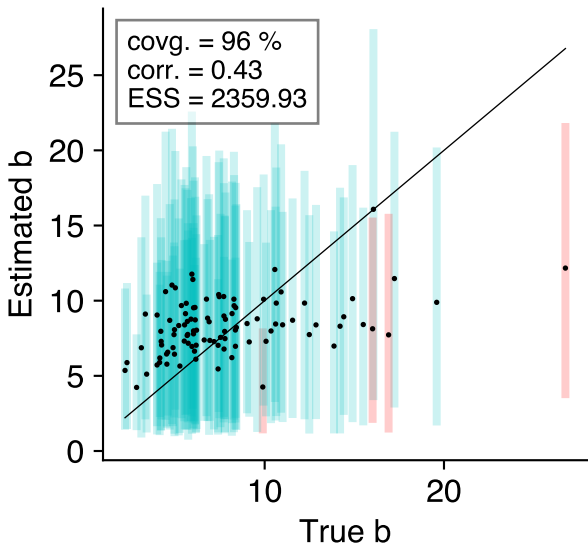

Supplement: msaf297_Supplementary_Data [file msaf297_supplementary_data.zip › Tex_LaTeX_suppl/supplement/figs/logisticExpansion/b.pdf]

NA

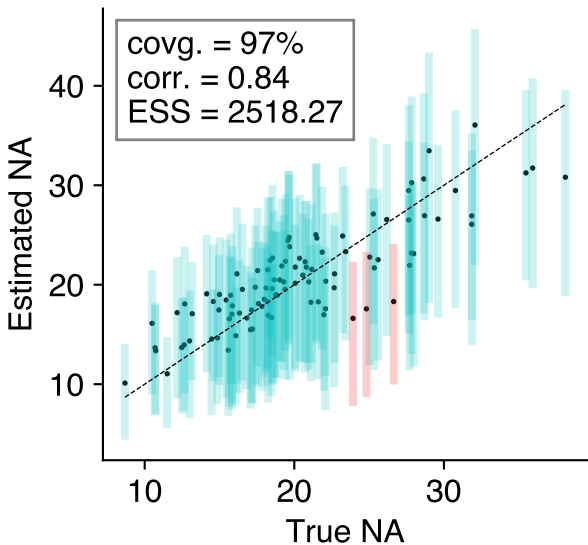

Supplement: msaf297_Supplementary_Data [file msaf297_supplementary_data.zip › Tex_LaTeX_suppl/supplement/figs/logisticExpansion/NA.pdf]

# nCarryingCapacity

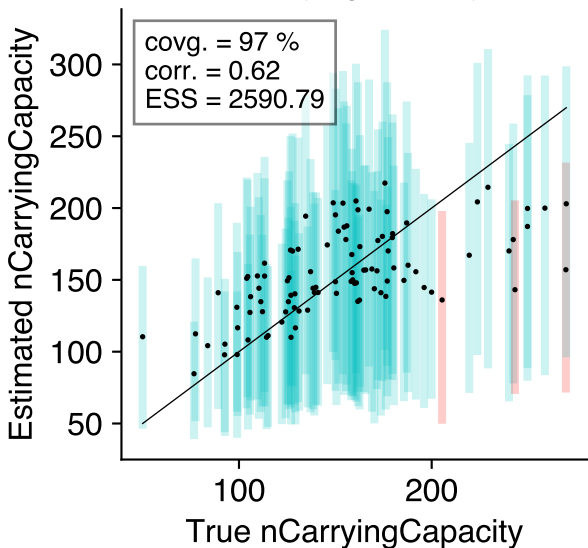

Supplement: msaf297_Supplementary_Data [file msaf297_supplementary_data.zip › Tex_LaTeX_suppl/supplement/figs/logisticExpansion/ncarryingcapacity.pdf]

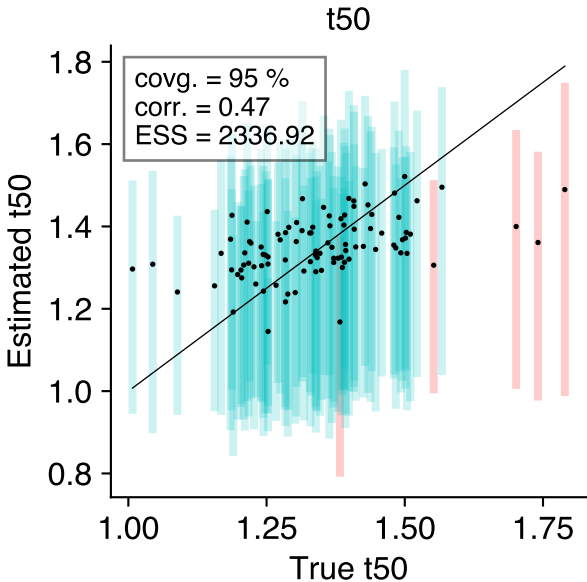

Supplement: msaf297_Supplementary_Data [file msaf297_supplementary_data.zip › Tex_LaTeX_suppl/supplement/figs/logisticExpansion/t50.pdf]

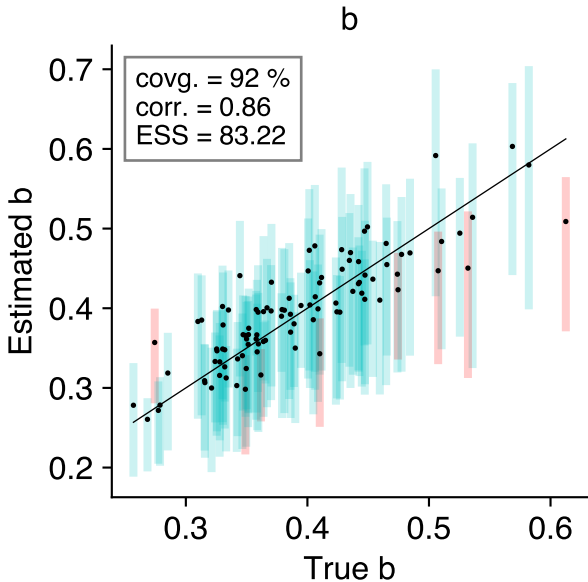

Supplement: msaf297_Supplementary_Data [file msaf297_supplementary_data.zip › Tex_LaTeX_suppl/supplement/figs/nooperator/f0/b.pdf]

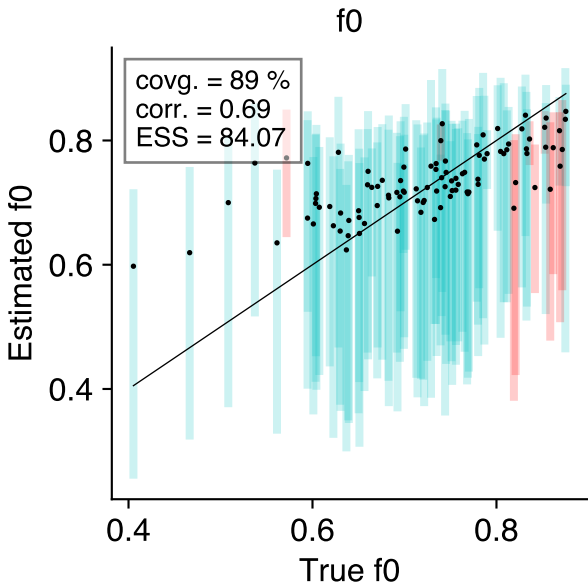

Supplement: msaf297_Supplementary_Data [file msaf297_supplementary_data.zip › Tex_LaTeX_suppl/supplement/figs/nooperator/f0/f0.pdf]

N0

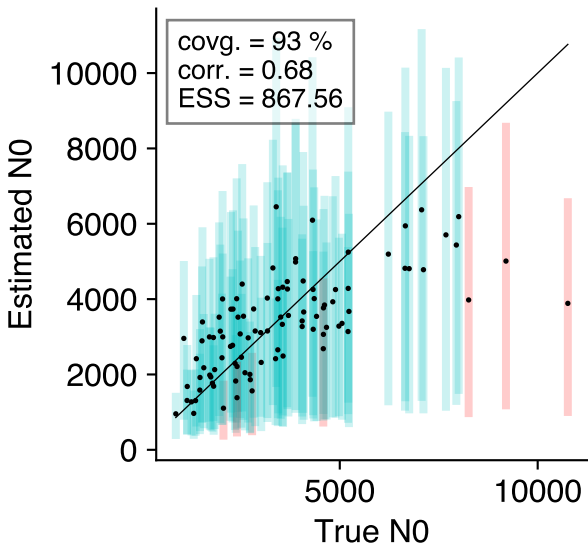

Supplement: msaf297_Supplementary_Data [file msaf297_supplementary_data.zip › Tex_LaTeX_suppl/supplement/figs/nooperator/f0/n0.pdf]

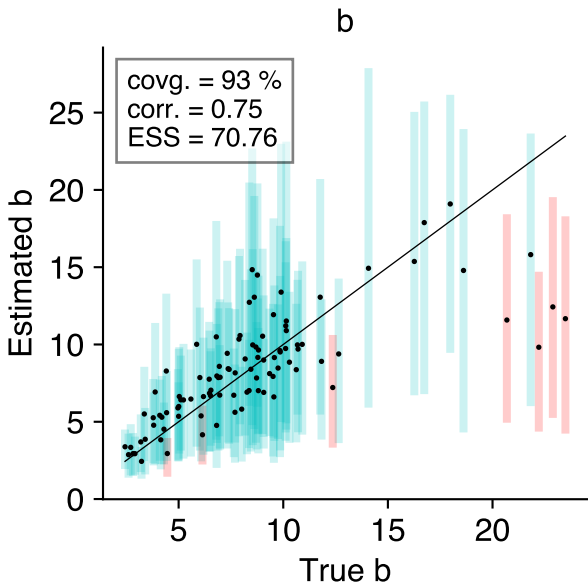

Supplement: msaf297_Supplementary_Data [file msaf297_supplementary_data.zip › Tex_LaTeX_suppl/supplement/figs/nooperator/t50/b.pdf]

# NInfinity

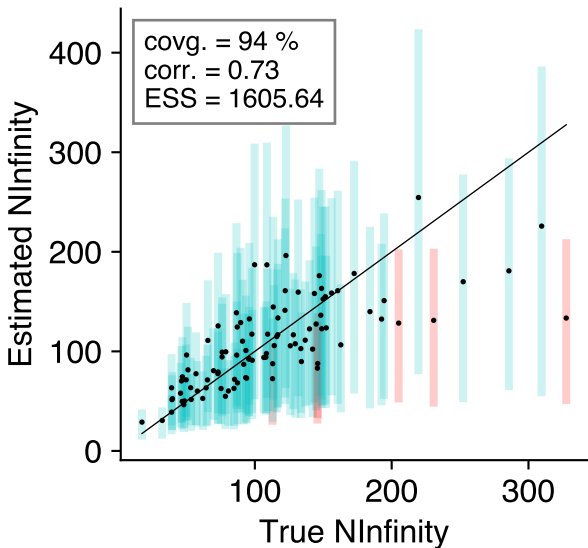

Supplement: msaf297_Supplementary_Data [file msaf297_supplementary_data.zip › Tex_LaTeX_suppl/supplement/figs/nooperator/t50/Ninf.pdf]

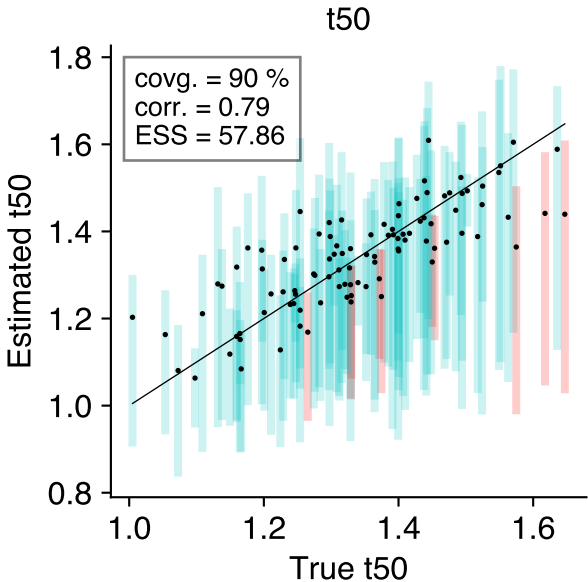

Supplement: msaf297_Supplementary_Data [file msaf297_supplementary_data.zip › Tex_LaTeX_suppl/supplement/figs/nooperator/t50/t50.pdf]

b

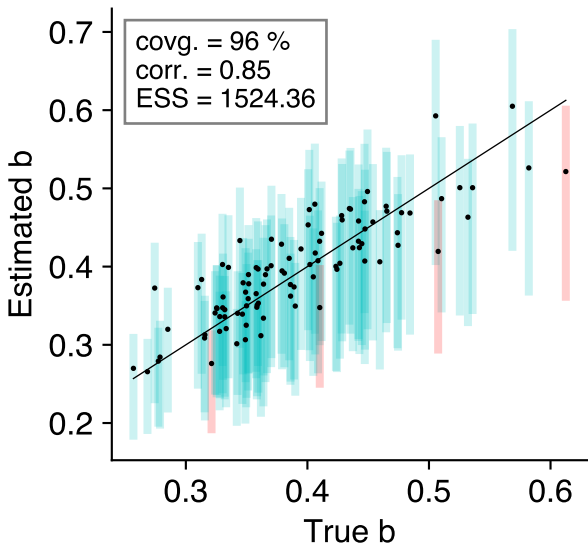

Supplement: msaf297_Supplementary_Data [file msaf297_supplementary_data.zip › Tex_LaTeX_suppl/supplement/figs/operator/f0/b.pdf]

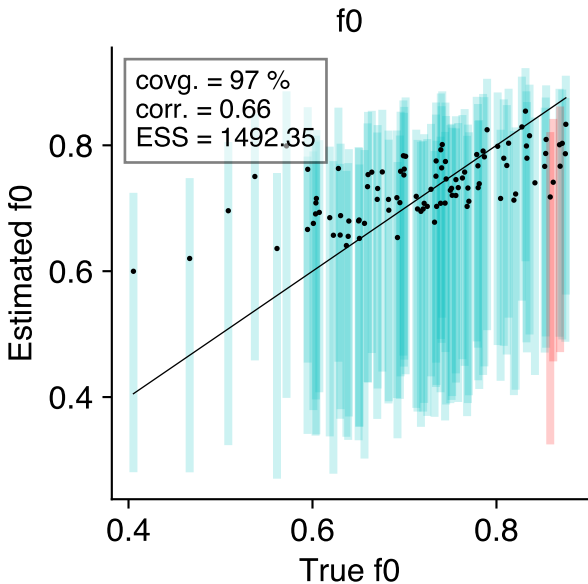

Supplement: msaf297_Supplementary_Data [file msaf297_supplementary_data.zip › Tex_LaTeX_suppl/supplement/figs/operator/f0/f0.pdf]

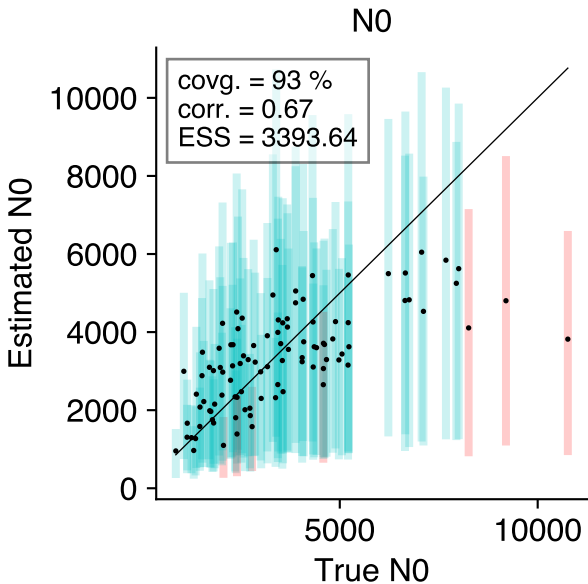

Supplement: msaf297_Supplementary_Data [file msaf297_supplementary_data.zip › Tex_LaTeX_suppl/supplement/figs/operator/f0/n0.pdf]

b

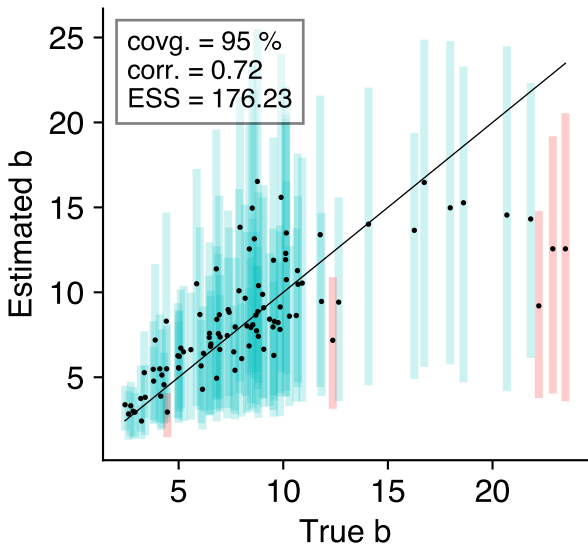

Supplement: msaf297_Supplementary_Data [file msaf297_supplementary_data.zip › Tex_LaTeX_suppl/supplement/figs/operator/t50/b.pdf]

# NlInfinity

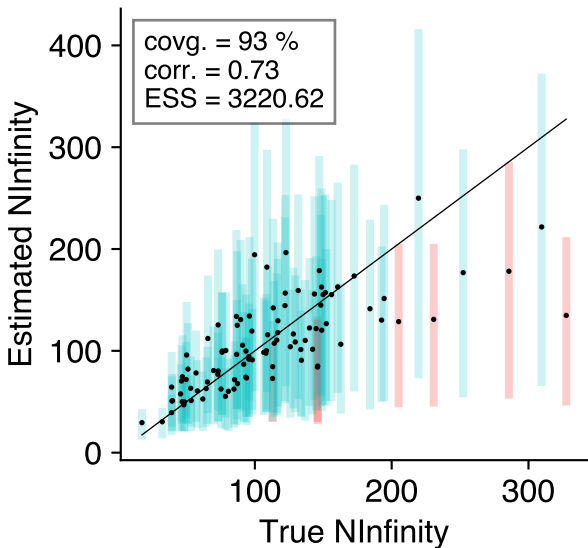

Supplement: msaf297_Supplementary_Data [file msaf297_supplementary_data.zip › Tex_LaTeX_suppl/supplement/figs/operator/t50/Ninf.pdf]

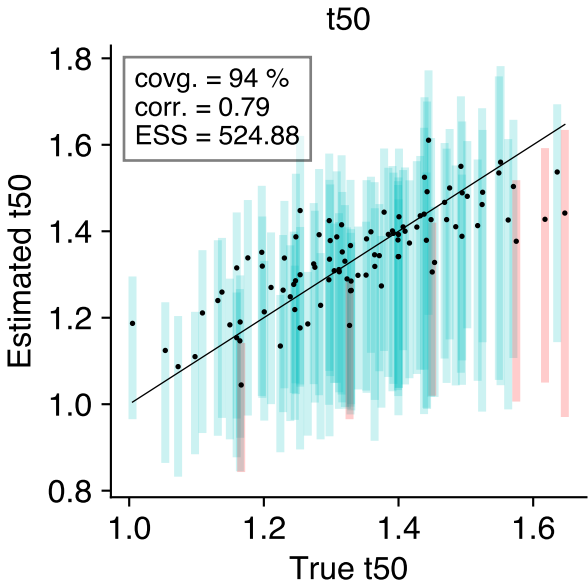

Supplement: msaf297_Supplementary_Data [file msaf297_supplementary_data.zip › Tex_LaTeX_suppl/supplement/figs/operator/t50/t50.pdf]
